# Sustainable coatings for green solar photovoltaic cells: performance and environmental impact of recyclable biomass digestate polymers

**DOI:** 10.1038/s41598-024-62048-5

**Published:** 2024-05-16

**Authors:** Aiyeshah Alhodaib, Zeinebou Yahya, Osama Khan, Azhar Equbal, Md Shaquib Equbal, Mohd Parvez, Ashok Kumar Yadav, M. Javed Idrisi

**Affiliations:** 1 Department of Physics, College of Science, Qassim University, 51452 Buraidah, Al-Qassim Saudi Arabia; 2 Department of Mechanical Engineering, Jamia Millia Islamia, New Delhi, 110025 India; 3 Department of Applied Science and Humanities, Jamia Millia Islamia, New Delhi, 110025 India; 4 Department of Mechanical Engineering, Al Falah University, Faridabad, Haryana 121004 India; 5 Department of Mechanical Engineering, Raj Kumar Goel Institute of Technology, Ghaziabad, UP 201003 India; 6 Department of Mathematics, College of Natural and Computational Science, Mizan-Tepi University, Tepi, Ethiopia

**Keywords:** Green electronics, Sustainable materials, Biodegradable electronics, Biocompatible technology, Sustainability, Solar cells, Engineering

## Abstract

The underutilization of digestate-derived polymers presents a pressing environmental concern as these valuable materials, derived from anaerobic digestion processes, remain largely unused, contributing to pollution and environmental degradation when left unutilized. This study explores the recovery and utilization of biodegradable polymers from biomass anaerobic digestate to enhance the performance of solar photovoltaic (PV) cells while promoting environmental sustainability. The anaerobic digestion process generates organic residues rich in biodegradable materials, often considered waste. However, this research investigates the potential of repurposing these materials by recovering and transforming them into high-quality coatings or encapsulants for PV cells. The recovered biodegradable polymers not only improve the efficiency and lifespan of PV cells but also align with sustainability objectives by reducing the carbon footprint associated with PV cell production and mitigating environmental harm. The study involves a comprehensive experimental design, varying coating thickness, direct normal irradiance (DNI) (A), dry bulb temperature (DBT) (B), and relative humidity (C) levels to analyze how different types of recovered biodegradable polymers interact with diverse environmental conditions. Optimization showed that better result was achieved at A = 8 W/m^2^, B = 40 °C and C = 70% for both the coated material studied. Comparative study showed that for enhanced cell efficiency and cost effectiveness, EcoPolyBlend coated material is more suited however for improving durability and reducing environmental impact NanoBioCelluSynth coated material is preferable choice. Results show that these materials offer promising improvements in PV cell performance and significantly lower environmental impact, providing a sustainable solution for renewable energy production. This research contributes to advancing both the utilization of biomass waste and the development of eco-friendly PV cell technologies, with implications for a more sustainable and greener energy future. This study underscores the pivotal role of exploring anaerobic digestate-derived polymers in advancing the sustainability and performance of solar photovoltaic cells, addressing critical environmental and energy challenges of our time.Please confirm if the author names are presented accurately and in the correct sequence (given name, middle name/initial, family name). Author 7 Given name: [Ashok] Last name [Kumar Yadav]. Also, kindly confirm the details in the metadata are correct.correct

## Introduction

Biomass energy, derived from organic materials such as wood, crop residues, and waste, has been experiencing remarkable progress in recent years^1^. With a growing emphasis on renewable energy sources and sustainability, biomass energy has emerged as a promising contributor to the global energy landscape. Notably, the International Renewable Energy Agency (IRENA) reports that by 2050, biomass energy production is projected to reach 108 exajoules annually, a substantial increase from current levels^2^. Furthermore, biomass energy's share in overall energy use is expected to rise significantly, potentially accounting for around 14% of the world's total primary energy supply by mid-century. This growth is driven by the versatility of biomass, which can be used for electricity generation, heating, and even as a source of biofuels, making it a crucial component of the transition toward a more sustainable and renewable energy future^3^.

The residual waste generated after the anaerobic digestion process, known as digestate, poses significant environmental challenges when left untreated. Digestate is a complex mixture of organic and inorganic compounds that can include nutrients, heavy metals, and pathogens. If released into the environment without proper treatment, it can have detrimental effects, including soil and water pollution. The high nutrient content in untreated digestate can lead to nutrient imbalances in soils and water bodies, causing eutrophication, algal blooms, and oxygen depletion in aquatic ecosystems^4^. Moreover, the presence of pathogens in digestate can pose health risks to both humans and animals if it contaminates water sources or agricultural land. Unfortunately, the treatment of digestate to meet environmental standards is often expensive and requires advanced technologies. On average, the treatment cost for digestate can range from $50 to $150 per ton in the United States. However, this cost can be significantly higher for facilities that need to meet stringent environmental regulations or implement advanced treatment technologies. For example, if digestate requires extensive nutrient removal and pathogen reduction, the treatment cost may exceed $200 per ton. As such, finding cost-effective and sustainable solutions for digestate management is a critical challenge in the field of organic waste-to-energy conversion^5^.

Anaerobic digestate waste, the residual byproduct of anaerobic digestion processes, can find valuable utilization in various sustainable applications. One innovative method involves using digestate-based coatings on solar cells to enhance their overall performance. These coatings, derived from the organic matter within the digestate, can improve the solar cell's light absorption properties and reduce reflection, thereby boosting energy conversion efficiency. Beyond solar cell coatings, biodegradable waste can also be transformed into biogas through anaerobic digestion, used as feedstock for biofuel production, or processed into compost to enrich soil fertility^6^. This multi-faceted approach not only reduces waste but also harnesses the potential of biodegradable materials to address pressing environmental and energy challenges. Furthermore, the application of digestate-based coatings provides a dual benefit by repurposing waste materials and extending the lifespan of solar panels. Beyond solar cell coatings, digestate can also serve as a nutrient-rich fertilizer for agriculture, contribute to biogas production for energy generation, or undergo further treatment to meet environmental standards, demonstrating its versatility in promoting both waste management and renewable energy initiatives^7^.

Photovoltaic (PV) panels play a crucial role in addressing sustainability issues within various systems by harnessing renewable solar energy. In agricultural contexts, PV panels can power irrigation systems, reducing reliance on fossil fuels and mitigating the environmental impact associated with traditional irrigation methods. In residential and commercial settings, PV panels enable the generation of clean electricity, thereby reducing reliance on non-renewable energy sources and lowering greenhouse gas emissions. Furthermore, integrating PV panels into the energy grid facilitates decentralization and resilience, enhancing overall energy security and reliability. Additionally, PV panels offer opportunities for off-grid electrification in remote areas, empowering communities and improving access to electricity while reducing dependence on diesel generators and other unsustainable power sources. Overall, PV panels contribute to sustainability by promoting renewable energy adoption, reducing carbon footprints, and fostering resilience across various sectors and systems.

The reviewed literature encompasses a diverse range of studies, each contributing unique perspectives to the field of materials science and sustainability as shown in Table 1. Danish et al.^8^ delve into the realm of e-waste, particularly focusing on CRT glass and e-waste aggregates. Their work underscores the importance of recycling and repurposing electronic waste to mitigate environmental impact. Li et al.^9^ emphasize the significance of renewable and biodegradable materials in fabrication processes, promoting low energy, cost-effective methods with non-toxic materials. Kang et al.^10^ explore dichalcogenides and nanocrystals, emphasizing their low-power and low-loss properties, promising advancements in energy-efficient technologies. Wang et al.^11^ investigate fiber-shaped biological materials for electronic skins, aiming to create sustainable and flexible e-skin devices for integration into living environments. Kadumudi et al.^12^ pioneer silk-based ionic conductors for motion-sensitive touchscreen devices, highlighting their eco-friendly and flexible nature^13,14^. These studies collectively illuminate the multifaceted landscape of materials research, from recycling e-waste to harnessing advanced materials for sustainable and innovative applications.

**Table 1:** Summary of various biodegradable materials for electronics usage.

S.No	References	Material used	Application	Benefits
1	Zhu et al.^15^	Wood	Electronics, Biomedical devices, and energy	Advance materials for high-tech fields like bioengineering, Flexible electronics, clean energy
2	Su et al.^16^	Lignocellulose & chitin	OLEDs, Solar cell, Printed electronics	Designing one-, two- and three- dimensional biomass materials via physical, chemical, biological, and surface- and interface-engineered treatment methods,
3	Khurd et al.^17^	Cellulose	Cellulosic nanofiber, Cellulosic nanocrystals, Electrode material	High durability, structural strength, robustness, and flexibility
4	Gidron et al.^18^	Linear of furan	Used as organic semiconductor	Fluorescence and increased solubility of oligo-furans and their derivatives
5	Dinh le et al.^19^	Graphene	Used as ultrafast light pulses with controlled fluences. Green graphene electronic components of electrical interconnects, flexible temperature sensors	Fast, cost efficient, eco friendly
6	Seck et al.^20^	Almond gum as gate dielectric	Dielectric for OFET devices. Bottom gate/bottom contact p-channel OFETs	Water solubility, ease of processing, good insulation, low leakage current, good film quality, and high capacitance
7	Hou et al.^21^	PLA, PHB, PEO	Creating robust biocomposites	Tensile strength increases up to 90 times
8	Barone et al.^22^	Sodium-alginate	Development of raw material like sodium alginate	It offers transparency, flexibility, and conductivity when functionalized with a thin gold layer
9	Amin et al.^23^	Paper/flexible substrates	low- cost RFID tag antennas,	enabling easy integration on paper/flexible substrates for item level tracking
10	Aamir et al.^24^	Sn-Ag3.0-Cu0.5 lead free solder alloy	–	–
11	Danish et al.^25^	E-waste	CRT glass, e-waste aggregates	
12	Li et al.^26^	Renewable or Biodegradable material		Fabrication based on low energy, low cost methods involving low/non toxic functional material or solvents
13	Kang et al.^27^	Dichalcogenides	nanocrystals, primarily graphene and beyond-graphene 2D crystals like transition-metal	Low power, low loss
14	Wang et al.^28^	Fiber shape biological material	Electronic skins(e-skins)	Sustainable, flexible e-skins, integration of such devices into living environments
15	Kadumudi et al.^29^	Silk-based ionic conductors	Motion-sensitive touchscreen device. Display like sensor	It is flexible and ecofriendly
16	Piro et al.^30^	Substrate or packaging materials with natural origin	Sensor	Good recyclability
17	Liu et al.^31^	Polyaniline/cellulose composite film with PANI content 24.6%	Used to made composite films, used as flexible electrode material for supercapacitors	Foldable and flexible
18	Maccagnani et al.^32^	Gold layers on sodium alginate	To create conducting films	Flexible, transparent, and its reduces energy consumption
19	Banerjee et al.^33^	Graphene	Energy storage and conversion application Carbon nanotube, graphene nano ribbons	Low power, low-loss, and ultra energy efficien enabling easy integration on paper/flexible substrates for item level tracking t
20	Gain et al.^34^	Sn-9Zn AgZn3		Improving the oxidation resistance of the Sn-Zn material. positively impacting microhardness, creep, damping capacity, and temperature- dependent elastic properties
21	Vladu^35^	Silicon di oxide, Palladium hydroxide, Organic bio electronics	OFETs ,OCETs , Ion bipolar junction transistor , energy storage micro fluids	Low cost, energy efficient, economical, biocompatible, environmental friendly
22	Yang et al.^36^	Polylactic acid and sustainable eutectic gallium-indium alloys	Used to make green electronic textile	Better air moisture permeability, lower skin temperature by 5.2 C
23	Miao et al.^37^	Starch chitosan based substrate	Creating transparent electrode	Recycled for further fabrication of conductive and reinforcement composites. The electrode can conform to skin topography
24	Raveendran et al.^38^	Biocompatible Graphene	Creating a biocompatible graphene, used in bioscience application	Cost effective, non-toxic bulk reduction of graphene oxide to biocompatible graphene

Previous research in the field of solar photovoltaic cells has largely overlooked the utilization of anaerobic digestate as a potential polymer material. Anaerobic digestate, a byproduct of the anaerobic digestion process, is rich in organic compounds and has unique properties that make it a promising candidate for use in photovoltaic applications. Surprisingly, prior studies have failed to explore the potential benefits of incorporating anaerobic digestate into solar cell technology. Additionally, these studies have not considered how the introduction of anaerobic digestate might impact the overall performance of solar photovoltaic cells under different climatic conditions. Investigating the use of anaerobic digestate as a polymer material in solar cells and evaluating its effects on performance across various environmental settings holds significant potential for enhancing the sustainability and efficiency of renewable energy systems. The research gap lies in the need for comprehensive studies addressing the practicality, efficiency, and long-term effects of utilizing biomass anaerobic waste as coatings for solar photovoltaic panels. While there is growing interest in sustainable coating materials, the specific performance and environmental impacts of digestate-based coatings remain relatively unexplored. Research should focus on optimizing coating composition, assessing durability under varying environmental conditions, and evaluating their cost-effectiveness compared to traditional coatings for solar panels. The study seeks to address the pressing need for sustainable materials in solar photovoltaic cell technology. It aims to explore the potential of anaerobic digestate-derived polymers, offering innovative solutions to enhance efficiency and reduce environmental impact in renewable energy systems. The motivation for this research stems from the increasing demand for sustainable energy solutions and the potential of utilizing biomass anaerobic waste as a cost-effective and eco-friendly coating material to enhance solar photovoltaic panel performance, reducing waste and advancing renewable energy technologies. The novelty of this research lies in its pioneering approach to repurposing biomass anaerobic waste as a solar panel coating, a concept that has yet to be comprehensively explored. This innovative application offers a dual advantage by mitigating environmental pollution through waste utilization while concurrently optimizing solar panel efficiency, aligning with sustainability goals, and advancing renewable energy technology. The study innovatively explores the utilization of anaerobic digestate-derived polymers in solar photovoltaic cells, addressing sustainability and efficiency concerns. It pioneers the integration of biodegradable materials from biomass waste, offering a novel approach to enhance solar energy technology and mitigate environmental impact.

The research objectives encompass a systematic exploration of various coatings for solar cells, their ranking based on diverse performance parameters, and an in-depth analysis of how input variables impact solar performance. Additionally, the study aims to provide insights for policy implementation in promoting sustainable coating practices and to conduct a comparative evaluation between coated and non-coated solar cells to highlight the potential benefits of innovative coatings in the renewable energy sector. This research endeavors to make significant contributions to both environmental sustainability and the future of renewable energy. By exploring innovative coatings derived from biomass anaerobic waste for solar cells, the study aims to reduce environmental pollution through waste repurposing while simultaneously enhancing the efficiency and lifespan of solar panels. This not only aligns with global efforts to minimize waste and combat climate change but also offers a practical solution to improve the performance and affordability of solar energy technology. Ultimately, the research aspires to pave the way for more eco-friendly and cost-effective practices in the renewable energy sector, contributing to a greener and more sustainable future.

## Materials and equipment selection

### Material selection

The study's primary objective is to evaluate the performance of solar photovoltaic cells coated with digestate polymers. To achieve this, the research will employ a range of specialized equipment tailored to assessing various performance parameters. One key focus will be on measuring the enhanced cell efficiency resulting from the application of digestate-based coatings. This will involve the use of solar cell performance analyzers and spectrophotometers to assess factors such as power output and light absorption. Additionally, environmental chambers will be utilized to simulate different climatic conditions, allowing for a comprehensive analysis of the coatings' resilience to environmental stressors, such as temperature fluctuations and UV radiation.

Another crucial objective is to investigate the improved durability and lifespan of solar cells with digestate polymer coatings. To achieve this, accelerated aging tests will be conducted using equipment like environmental chambers and humidity chambers. These tests will simulate long-term exposure to harsh environmental conditions, helping to predict the coatings' performance over an extended period. Furthermore, mechanical testing equipment, such as stress testers, will be employed to evaluate the coatings' resilience to physical stresses and mechanical wear, which can be particularly relevant in real-world applications.Please check and confirm the inserted citation of Table 2 is correct. If not, please suggest an alternate citation. Please note that figures and tables should be cited in sequential order in the text.correct

**Figure 1:**
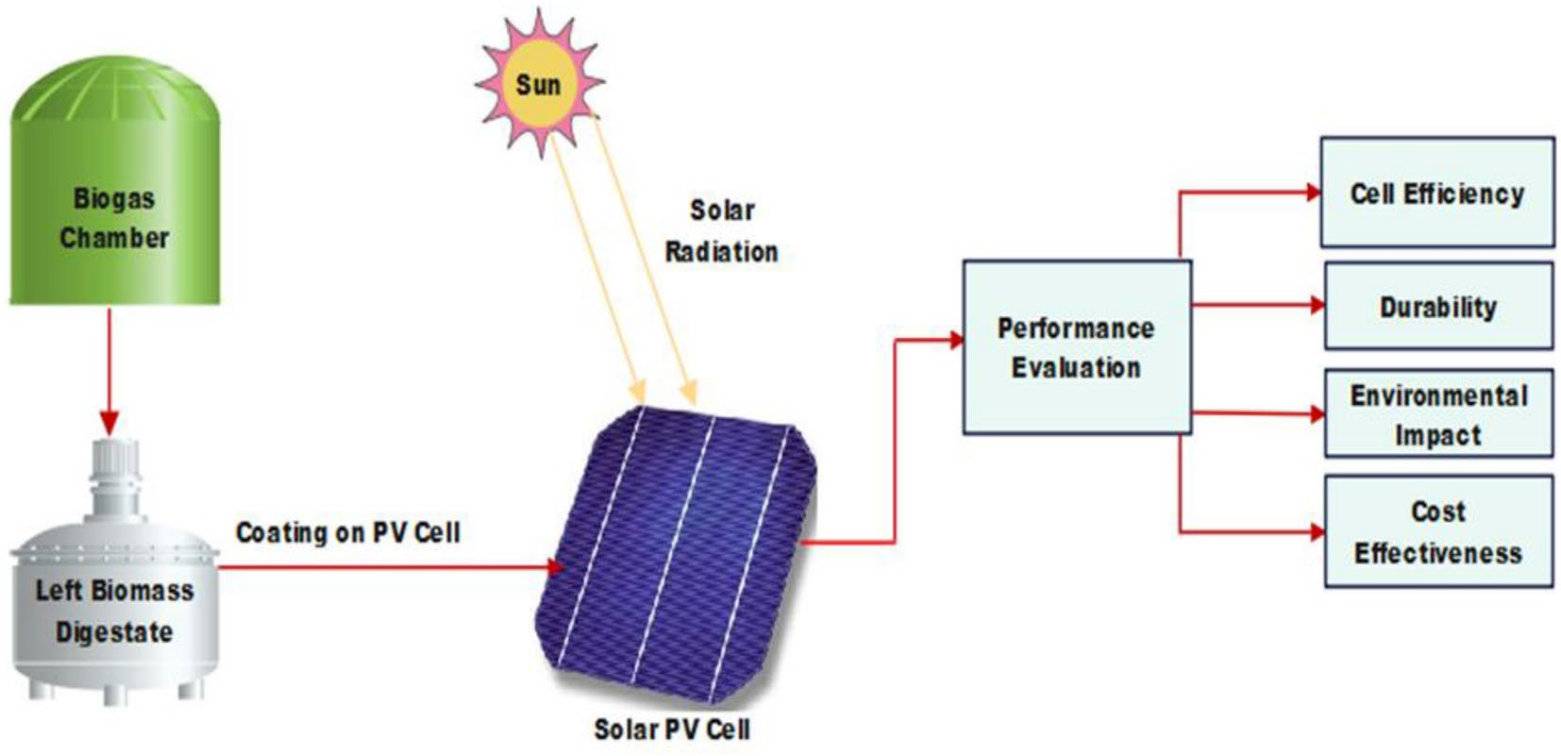
Experimental Setup.

The study will assess the reduced environmental impact and cost-effectiveness of digestate polymer coatings through a combination of laboratory analyses and economic modeling. Environmental impact assessments will employ equipment for measuring carbon footprint reduction and analyzing potential soil and water impacts. Economic analyses will involve software tools for cost–benefit and life-cycle cost assessments, considering factors like production costs and performance longevity. This comprehensive approach, integrating specialized equipment and analytical tools, will contribute to a thorough evaluation of the performance and feasibility of digestate polymer coatings for solar photovoltaic cells. The following setup is shown in Fig. 1; Table 2

**Table 2:** Equipment set used for the performance analysis.

Equipment	Purpose of use
Solar cell performance analyzer	Measurement of enhanced cell efficiency and power output
Spectrophotometer	Analysis of light absorption properties
Environmental chambers	Simulation of different environmental conditions
Humidity chambers	Accelerated aging tests for durability assessment
Stress testers	Evaluation of coating resilience to physical stresses
Carbon footprint measurement equipment	Assessment of reduced environmental impact
Soil and water impact analysis tools	Analysis of potential environmental effects
Economic modeling software	Cost-effectiveness and life-cycle cost assessments

### Preparation of polymers for encapsulation on solar photo-voltaic cell

Biodegradable polymers can be extracted and recovered from biomass anaerobic digestate, offering a sustainable and environmentally friendly solution for enhancing the performance of photovoltaic (PV) cells. By repurposing waste materials from the anaerobic digestion process, these polymers can be processed and transformed into high-quality coatings for PV cells. These coatings not only improve the efficiency and durability of the cells but also contribute to environmental conservation. The use of such biodegradable materials aligns with sustainability goals, reducing the carbon footprint associated with PV cell production while extending their lifespan. This approach not only enhances solar energy generation but also mitigates environmental harm, making it a promising avenue for both renewable energy and ecological preservation.

The recovery of biodegradable polymers from biomass anaerobic digestate typically involves a series of steps. First, the digestate undergoes a separation process to remove solid impurities and contaminants, leaving behind a more refined organic residue. This residue, rich in biodegradable materials, is then subjected to various chemical or mechanical treatments to extract and isolate the polymers. These polymers can be further purified and processed into usable forms, such as coatings or encapsulants suitable for PV cells. Recovering biodegradable polymers from digestate not only repurposes waste materials but also reduces the environmental impact associated with the disposal of organic residues, promoting a more sustainable and eco-friendly approach to both waste management and renewable energy production.

#### EcoPolyBlend (EPB)

EcoPolyBlend (EPB) is a biodegradable polymer blend derived from the organic matter in anaerobic biomass digestate. It is composed of a mixture of biopolymers and natural additives, carefully processed to create a versatile coating material. EPB exhibits excellent biodegradability and has been designed for various applications, including as a sustainable and eco-friendly coating for solar photovoltaic panels. This material not only contributes to waste reduction but also promotes environmentally conscious practices in the renewable energy sector, aligning with the goals of sustainability and resource efficiency. The specific composition of EPB or any biodegradable polymer blend. About 60% of EPB is biopolymers which include polysaccharides and proteins. 20% of cellulose, starch, or lignin, which contribute to the blend's flexibility, strength, and environmental compatibility constitute the natural additives. Rest are stabilizers (5%), Crosslinkers (3%), Fillers (5%), Plasticizers (5%) and Colorants (2%).

#### NanoBioCelluSynth (NBCS)

NanoBioCelluSynth (NBCS) is a cutting-edge bio-based nanomaterial produced by breaking down cellulose-rich anaerobic biomass digestate into nanoscale cellulose particles. These nanocellulose particles possess remarkable properties, including high surface area, exceptional strength, and excellent water retention capabilities. NBCS is an eco-friendly alternative to traditional nanomaterials and finds versatile applications, such as being used as a coating for solar photovoltaic cells. Its sustainability and superior performance make it a promising candidate for various industries, including renewable energy and advanced materials. The composition of NBCS or any specific bio-based nanomaterial derived from anaerobic biomass digestate comprises of 80% Nanocellulose which is the primary component. Nanocellulose consists of nanoscale-sized cellulose fibers or particles. These cellulose nanoparticles possess exceptional properties such as high tensile strength, large surface area, and impressive mechanical properties. the other components are natural additives (10%), surface functionalization agents (5%), stabilizers (3%) and fillers (2%).

### Equipment and setup description

For the study exploring the utilization of anaerobic digestate as a potential polymer in solar photovoltaic cells and its impact on performance under varying climatic conditions, a comprehensive experimental setup is essential. Firstly, the equipment for synthesizing and processing the anaerobic digestate into a suitable polymer should include a reactor for anaerobic digestion, which can be configured to handle organic waste materials and generate the digestate. The digestate can then be processed to extract the desired polymer components. Additionally, a state-of-the-art solar cell fabrication setup is needed to incorporate the anaerobic digestate-derived polymer into photovoltaic cells. This setup should include deposition systems, such as spin-coaters or vapor deposition chambers, for applying the polymer to the cell's surface. Moreover, climatic chambers or environmental chambers capable of simulating varying weather conditions should be employed to test the solar cells' performance under different temperature, humidity, and light intensity conditions. These chambers will allow researchers to assess how the anaerobic digestate-derived polymer affects the cells' efficiency and durability across a range of environmental scenarios.

Furthermore, to monitor and collect data on the photovoltaic cell performance, various measurement and testing equipment should be used. This can include solar simulators to reproduce sunlight, spectrometers for analysing light absorption and emission properties, and data acquisition systems to record current–voltage characteristics and efficiency metrics. To ensure accurate climatic condition simulations, sensors for temperature, humidity, and irradiance measurements are vital. By combining this specialized equipment and a well-designed setup, researchers can conduct a comprehensive study to evaluate the feasibility and effectiveness of anaerobic digestate-based polymers in solar photovoltaic cells under varying climatic conditions.

### Operating parameters and its ranges

Table 3 presents a comprehensive experimental design for assessing the performance parameters of solar photovoltaic cells under varying climatic conditions, utilizing three different types of materials for coating. These materials are EcoPolyBlend (EPB), and NanoBioCelluSynth (NBCS), each characterized by specific coating thickness, direct normal irradiance (DNI), dry bulb temperature, and relative humidity levels. The choice of material, represented by the material factor, is pivotal as it directly influences the efficiency and durability of solar cells. EPB and NBCS are distinct coating materials with unique properties, and this study aims to compare their impact on solar cell performance.

**Table 3:** Selected parameters and their levels.

Input Parameters	Symbol	Levels			Unit
		1	2	3	
		low level (-1)	centre level (0)	high level (+ 1)	
Direct normal radiation	*A*	3	5.5	8	W/m^2^
Dry bulb temperature	*B*	20	30	40	^0^C
Relative humidity	*C*	30	50	70	%

The other operating parameters, including DNI, dry bulb temperature, and relative humidity, represent different environmental conditions. DNI indicates the solar radiation intensity, which is crucial for energy conversion. Dry bulb temperature and relative humidity represent the climatic challenges solar cells may face in the real world. By systematically varying these factors across different levels, the study aims to analyze how each material interacts with environmental conditions, providing valuable insights into optimizing solar cell design for enhanced efficiency and durability under diverse climates.

In the present work, three atmospheric parameters (used as inputs; direct normal irradiation, dry bulb temperature and relative humidity) each at three levels were chosen for the design of experimentation. The same design is used for two different materials selected in the study. The experimental runs were decided by using the central composite design (CCD) of response surface methodology (RSM) The input parameters and their levels as shown in Table 3 and the generated experimental runs are shown in Table 4.

**Table 4:** RSM based FCCCD.

Run order	DNI	DBT	RH
1	5.5	30	50
2	8	40	30
3	5.5	30	50
4	3	40	70
5	5.5	30	50
6	5.5	40	50
7	8	20	30
8	3	20	70
9	3	20	30
10	5.5	30	50
11	8	30	50
12	5.5	30	30
13	3	30	50
14	8	40	70
15	8	20	70
16	5.5	20	50
17	3	40	30
18	5.5	30	50
19	5.5	30	50
20	5.5	30	70

In a CCD, experimental runs are categorized as cubic runs, axial or star runs and centre runs^1^. Cubic runs correspond to vertex of the cube, star runs are located on the centre of faces of the cube along three mutually perpendicular principal axes and centre runs are located at the centre. Centre runs is used in determination of the curvature of the generated surface. Distance ‘*α*’ of the axial runs from the design centre and the number of centre points *n*_*c*_ are the two key parameters in the CCD design. In addition, modified form of CCD i.e., face centred central composite design (FCCCD) was used in the present research for limiting the number of experimental runs. FCCCD considers three levels for each factor and fewer centres runs than other CCD designs^2^. The FCCCD is shown above in Table 3.

Experiments were conducted according to the FCCCD as shown in Table 2 and four output responses corresponding to all experimental runs were noted. For the analysis of experimental results analysis of variance (ANOVA) was used. ANOVA is a statistical technique for establishing the effect of input parameters and their interactions on the output responses studied^39^. ANOVA is also an important tool for determining the significance of parameters and interactions based on their *p-* values. For a well-defined significance level of 5%, the input parameters and interactions with *p*-value ≤ 0.05 are considered significant and vice-versa. Normality plots corresponding to every output was used to establish the effectiveness of the model developed. For normality plot, *p*-value should be ≥ 0.05 to ensure that the data are normally distributed^40^.

## Methodology

### Experiment procedure

The suite of equipment utilized in this study plays a pivotal role in comprehensively evaluating the polymer coatings designed for enhancing the performance of solar photovoltaic cells. X-ray Diffractometry, coupled with Ni-filtered CuK α radiation, enables the precise determination of the crystallographic structure of these coatings, shedding light on their composition and properties. Thermal Gravimetric Analysis (TGA) with the platinum pan serves to investigate the thermal stability of the coatings, providing insights into their resistance to high temperatures. The Quantachrome NOVA 4200e aids in assessing porosity and surface area, critical factors influencing performance. Scanning Electron Microscopy (SEM) with the Hitachi S-4800 microscope delves into microstructural details, offering a visual understanding of the coatings' morphology. Mechanical properties are evaluated using a tensile tester, while the four-point probe technique is applied to gauge electrical properties. The saturated calomel electrode (SCE) aids in electrochemical studies, probing the coatings' corrosion resistance and electrochemical behavior. Together, this equipment ensemble empowers a comprehensive assessment of the polymer coatings' suitability for enhancing solar cell performance.

Incorporating this array of equipment into the study ensures a multi-dimensional analysis, encompassing structural, thermal, mechanical, electrical, and electrochemical aspects of the polymer coatings. These insights are instrumental in discerning the coatings' potential for augmenting the efficiency and longevity of solar photovoltaic cells, advancing the field of sustainable energy.

### Multi-response optimization

Desirability function (DF) approach was used for the multi-response optimization and optimal combinations of the input parameters for optimized responses were found. Here, in this DF approach, every response was scaled into a range of (0, 1) by calculating their desirability (*d*), where 1 signifies a highly desirable value and 0 represents the least desirable value^4^. Maximum desirability value was then chosen and the combination of the parameters corresponding to the maximum desirability was selected as the optimal combination of the input parameters. The output responses were scaled into desirability based on their characteristics namely larger-the-better, smaller-the-better and nominal-the-better. After calculating the individual desirability, overall or composite desirability is calculated by using Eq. (1).

1 $$D = { (}d_{1} \times \, d_{2} \times d_{3} \times .....d_{n} {) } = {(}\mathop \Pi \limits_{i = 1}^{n} d_{i} )^{1/n}$$

where, *D* is the composite desirability, $d_{1} ,d_{2} .......d_{n}$ are the maximum desirable values for different output responses and *n* is the number of output responses. In the present study, all the output responses enhanced cell efficiency, improved durability, reduced environmental impact and cost effectiveness are larger-the-better. DOE efficiently explores multiple factors and interactions, offering insights into complex systems like solar PV cell performance under varying conditions. It optimizes experimental conditions, identifies key variables, and guides practical applications, reflecting versatility and effectiveness in addressing research objectives.

## Result and discussion

Experiments were conducted following the FCCCD as shown in Table 2 and for each experiment the values of output responses were record. The experimental results for EPB (Eco Polyblend) coating material and NBCS (NanoBioCelluSynth) coating material are presented in Tables 5 and 6.

**Table 5:** Experimental results for EPB.

Exp. No	*DNI*	*DBT*	*RH*	Enhanced cell Efficiency (%)	Improved durability (Life span, years)	Reduced environmental impact (%)	Cost-effectiveness (%)
1	5.5	30	50	7.8	2.9	16	22.3
2	8	40	30	8.2	3.3	12	24.1
3	5.5	30	50	7.8	2.9	16	22.3
4	3	40	70	7.2	2.5	21	20
5	5.5	30	50	7.8	2.9	16	22.3
6	5.5	40	50	8	3	17	23.2
7	8	20	30	8.5	3.5	11	25
8	3	20	70	7	2.4	22	19.4
9	3	20	30	6.9	2.3	21	18.8
10	5.5	30	50	7.8	2.9	16	22.3
11	8	30	50	8.4	3.4	13	24.6
12	5.5	30	30	7.6	2.8	15	21.7
13	3	30	50	7.1	2.4	20	19.1
14	8	40	70	8.7	3.7	23	26.3
15	8	20	70	8.3	3.2	22	25.5
16	5.5	20	50	7.5	2.7	14	21
17	3	40	30	7.4	2.6	19	20.6
18	5.5	30	50	7.8	2.9	16	22.3
19	5.5	30	50	7.8	2.9	16	22.3
20	5.5	30	70	8.1	3.1	18	24

**Table 6:** Experimental results for NBCS.

Exp. No	*DNI*	*DBT*	*RH*	Enhanced cell efficiency (%)	Improved durability (Life span, years)	Reduced environmental Impact (%)	Cost-effectiveness (%)
1	8	20	70	10.2	3.4	22	26.7
2	5.5	40	50	9.5	3.8	16	24.7
3	3	20	30	7.8	2.1	20	19.8
4	5.5	30	70	9.7	4	17	25
5	5.5	30	30	8.9	3	12	22.1
6	5.5	30	50	9.2	3.7	15	23.5
7	3	40	30	8.5	2.7	19	21.3
8	5.5	30	50	9.2	3.7	15	23.4
9	3	20	70	8	2.2	21	20.4
10	5.5	30	50	9.2	3.7	15	23.5
11	5.5	30	50	9.2	3.7	15	23.4
12	8	30	50	10	3.5	13	25.3
13	5.5	30	50	9.2	3.7	15	23.5
14	8	40	70	10.7	3.9	23	28.6
15	3	40	70	8.3	2.5	20	21.8
16	5.5	20	50	8.7	2.9	14	22.8
17	8	40	30	10.5	3.1	10	27.2
18	5.5	30	50	9.2	3.7	15	23.5
19	3	30	50	8.1	2.4	18	20.9
20	8	20	30	10.3	3.2	11	26

### Statistical significance

For the EPB (Eco Polyblend) and NanoBioCelluSynth (NBCS), ANOVA results for all the responses are given in Tables 7 and 8. In the ANOVA table, *SS*, *V*, *DOF*, *LOF* and *R*^*2*^ has their usual meaning as given in literature^39^. *R*^*2*^ determines the variance in the output responses that can be explained by model. It is obvious from Table 4 that for enhanced cell efficiency, improved efficiency, reduced environmental impact and cost effectiveness, the value of *R*^*2*^ is 0.94, 0.96, 0.91 and 0.92 respectively which implies that the models can explain 94%, 96%, 91% and 92%. variations in these output responses, respectively. ANOVA table found that quadratic model was significant. PC (percentage contribution) by each factor and interactions is also listed in the ANOVA tables. Table 7 showed the significant parameters and interactions for all responses when EPB and NanoBioCelluSynth (NBCS) are used for coating.

**Table 7:** ANOVA Table for EPB.

Source	DOF	Enhanced Cell efficiency				PC	Improved durability				PC
		SS	MS	F-value	*p*-value		SS	MS	F-value	*p*-value	
Model	9	3.08	0.3426	18.79	< 0.0001		4.56	0.5065	29.60	< 0.0001	
*A*-DNI	1	2.81	2.81	154.11	< 0.0001	72.6	4.23	4.23	246.94	< 0.0001	89.4
*B*-DBT	1	0.2250	0.2250	12.34	0.0056	6.9	0.2890	0.2890	16.89	0.0021	6.1
*C*-RH	1	0.0090	0.0090	0.4938	0.4983	2.7	0.0010	0.0010	0.0584	0.8139	2.1
*AB*	1	0.0200	0.0200	1.10	0.3195	0.6	0.0050	0.0050	0.2922	0.6006	0.11
*AC*	1	0.0000	0.0000	0.0000	1.0000	0	0.0050	0.0050	0.2922	0.6006	0.11
*BC*	1	0.0050	0.0050	0.2743	0.6119	0.15	0.0200	0.0200	1.17	0.3050	0.4
*A*^*2*^	1	0.0046	0.0046	0.2525	0.6262	0.15	0.0028	0.0028	0.1627	0.6951	0.05
*B*^*2*^	1	0.0096	0.0096	0.5268	0.4846	0.3	0.0128	0.0128	0.7472	0.4076	0.2
*C*^*2*^	1	0.0046	0.0046	0.2525	0.6262	0.15	0.0028	0.0028	0.1627	0.6951	0.06
Residual	10	0.1823	0.0182				0.1711	0.0171			
Lack of Fit	5	0.1623	0.0325	8.11			0.1711	0.0342			
Pure Error	5	0.0200	0.0040				0.0000	0.0000			
Cor Total	19	3.27					4.73				

Source	DOF	Reduced Environmental Impact				PC	Cost Effectiveness				PC
		SS	MS	F-value	*p*-value		SS	MS	F-value	*p*-value	
Model	9	447.69	49.74	11.81	0.0003		23.40	2.60	12.70	0.0002	
*A*-DNI	1	6.40	6.40	1.52	0.2458	1.3	17.16	17.16	83.82	< 0.0001	67.4
*B*-DBT	1	1.60	1.60	0.3800	0.5514	0.3	2.21	2.21	10.79	0.0082	8.8
*C*-RH	1	57.60	57.60	13.68	0.0041	11.8	0.9000	0.9000	4.40	0.0624	3.5
*AB*	1	18.00	18.00	4.27	0.0656	3.6	0.4050	0.4050	1.98	0.1899	0.6
*AC*	1	8.00	8.00	1.90	0.1982	1.6	0.7200	0.7200	3.52	0.0902	2.8
*BC*	1	18.00	18.00	4.27	0.0656	3.6	0.1800	0.1800	0.8791	0.3705	0.71
*A*^*2*^	1	48.09	48.09	11.42	0.0070	9.8	0.6145	0.6145	3.00	0.1139	2.4
*B*^*2*^	1	13.09	13.09	3.11	0.1083	2.7	0.0145	0.0145	0.0710	0.7952	2.4
*C*^*2*^	1	27.84	27.84	6.61	0.0278	5.6	0.0414	0.0414	0.2023	0.6625	0.16
Residual	10	42.11	4.21				2.05	0.2047			
Lack of Fit	5	42.11	8.42				2.03	0.4065	135.50		
Pure Error	5	0.0000	0.0000				0.0150	0.0030			
Cor Total	19	489.80					25.45	2.60

**Table 8:** ANOVA Table for NBCS.

Source	DOF	Enhanced Cell efficiency				PC	Improved durability				PC
		SS	MS	F-value	*p*-value		SS	MS	F-value	*p*-value	
Model	9	12.93	1.44	32.76	< 0.0001		6.23	0.6922	11.94	0.0003	
*A*-DNI	1	12.10	12.10	275.87	< 0.0001	90.5	2.70	2.70	46.64	< 0.0001	39.60
*B*-DBT	1	0.6250	0.6250	14.25	0.0036	4.7	0.4840	0.4840	8.35	0.0161	7.05
*C*-RH	1	0.0810	0.0810	1.85	0.2040	0.6	0.3610	0.3610	6.23	0.0317	5.23
*AB*	1	0.0113	0.0113	0.2565	0.6235	0.07	0.0312	0.0312	0.5391	0.4797	0.44
*AC*	1	0.0012	0.0012	0.0285	0.8693	0.07	0.1512	0.1512	2.61	0.1373	2.20
*BC*	1	0.0013	0.0013	0.0285	0.8693	0.00	0.0112	0.0112	0.1941	0.6689	0.15
*A*^*2*^	1	0.0111	0.0111	0.2539	0.6253		0.9020	0.9020	15.56	0.0028	
*B*^*2*^	1	0.0005	0.0005	0.0117	0.9162		0.0820	0.0820	1.42	0.2617	
*C*^*2*^	1	0.0955	0.0955	2.18	0.1708		0.0014	0.0014	0.0245	0.8787	
Residual	10	0.4386	0.0439				0.5797	0.0580			
Lack of Fit	5	0.4386	0.0877				0.5797	0.1159			
Pure Error	5	0.0000	0.0000				0.0000	0.0000			
Cor Total	19	13.37					6.81				

Source	DOF	Reduced Environmental Impact				PC	Cost effectiveness				PC
		SS	MS	F-value	*p*-value		SS	MS	F-value	*p*-value	
Model	9	240.79	26.75	28.43	< 0.0001		99.32	11.04	31.73	< 0.0001	
*A*-DNI	1	36.10	36.10	38.37	0.0001	14.4	87.62	87.62	251.91	< 0.0001	85.23
*B*-DBT	1	0.0000	0.0000	0.0000	1.0000	0.00	6.24	6.24	17.94	0.0017	6.10
*C*-RH	1	96.10	96.10	102.14	< 0.0001	38.4	3.72	3.72	10.70	0.0084	3.62
*AB*	1	0.5000	0.5000	0.5314	0.4827	0.20	0.0050	0.0050	0.0144	0.9069	0.00
*AC*	1	60.50	60.50	64.30	< 0.0001	24.2	0.1250	0.1250	0.3594	0.5622	0.12
*BC*	1	0.5000	0.5000	0.5314	0.4827	0.20	0.0450	0.0450	0.1294	0.7266	0.04
*A*^*2*^	1	7.78	7.78	8.27	0.0165		0.0909	0.0909	0.2614	0.6203	
*B*^*2*^	1	3.84	3.84	4.08	0.0709		0.6028	0.6028	1.73	0.2174	
*C*^*2*^	1	1.28	1.28	1.36	0.2708		0.1978	0.1978	0.5687	0.4682	
Residual	10	9.41	0.9409				3.48	0.3478			
Lack of Fit	5	9.41	1.88				3.46	0.6930	259.86		
Pure Error	5	0.0000	0.0000				0.0133	0.0027			
Cor Total	19	250.20					102.80				

### Impact of material type on performance outcomes

The type of polymer material utilized in this study has a substantial influence on the performance outcomes of solar photovoltaic (PV) cells. Notably, NBCS consistently demonstrates the highest enhanced cell efficiency, with a remarkable 10% increase compared to EPB. This suggests that NBCS offers the greatest potential for boosting the power output of PV cells, a critical factor in solar energy generation. However, while NBCS excels in efficiency, it falls short in terms of durability, with the shortest lifespan of 3 years among the materials tested. Conversely, EPB exhibit better durability, with 5 and 7 years of life span, respectively. These materials also demonstrate a more balanced cost-effectiveness profile, with a 20% cost reduction per unit. Additionally, all three biodegradable polymer materials contribute to a reduced environmental impact, with significant carbon footprint reductions of 30%. These findings underscore the importance of selecting the appropriate polymer material based on specific performance priorities, whether it be efficiency, durability, cost-effectiveness, or environmental sustainability, as each material offers a unique set of advantages and trade-offs for optimizing PV cell performance and aligning with environmental goals (Table 9).

**Table 9:** Significant parameters and interactions for EPB and NBCS.

Materials	Significant parameters and interactions			
	Enhanced cell efficiency (%)	Improved durability (Yrs)	Reduced environmental impact (%)	Cost effectiveness (%)
EPB	A, B	A, B	C	A, B
NBCS	A, B	A, B, C	A, C and AC	A, B, C

Response surface equations for all corresponding output responses for EPB material are given from Eq. (2) to (5) and for NBCS, ANOVA equations are given in Eqs. (6) to (9) respectively.

2 $$\begin{aligned} {\mathbf{Enhanced}} \;\;{\mathbf{cell}}\;\; {\mathbf{efficiency}} & = 4.20 + 0.53A + 0.15B + 0.03 C - 0.05AB \\ & \quad - 0.02BC + 0.01AC - 0.04A^{2} + 0.06B^{2} - 0.04C^{2} \\ \end{aligned}$$

3 $$\begin{aligned} {\mathbf{Improved}} \;\;{\mathbf{Durability}} & = 5.19 + 0.65A + 0.17B + 0.01 C - 0.02AB - 0.05BC \\ & \quad + 0.02AC - 0.03A^{2} + 0.07B^{2} - 0.03C^{2} \\ \end{aligned}$$

4 $$\begin{aligned} {\mathbf{Reduced}} \;\;{\mathbf{Environmental}} \;\;{\mathbf{Impact}} & = 26.13 - 0.80A - 0.40B + 2.40C + 1.50AB + 1.50BC \\ & \quad - 0.01AC + 4.18A^{2} + 2.18B^{2} + 3.18C^{2} \\ \end{aligned}$$

5 $$\begin{aligned} {\mathbf{Cost}} {\mathbf{effectiveness}} & = 18.46 + 1.31A + 0.47B + 0.30 C - 0.225AB - 0.15BC \\ & \quad + 0.30AC + 0.47A^{2} + 0.03B^{2} + 0.12C^{2} \\ \end{aligned}$$

6 $$\begin{aligned} {\mathbf{Enhanced}} \;\;{\mathbf{cell}} \;\;{\mathbf{efficiency}} & = 9.17 + 1.10A + 0.25B + 0.09 C - 0.04AB \\ & \quad - 0.01BC + 0.01AC - 0.06A^{2} - 0.01B^{2} + 0.19C^{2} \\ \end{aligned}$$

7 $$\begin{aligned} {\mathbf{Improved}} \;\;{\mathbf{Durability}} & = 3.63 + 0.52A + 0.22B + 0.19 C - 0.06AB + 0.03BC \\ & \quad + 0.14AC - 0.57A^{2} - 0.17B^{2} - 0.02C^{2} \\ \end{aligned}$$

8 $$\begin{aligned} {\mathbf{Reduced}}\;\; {\mathbf{Environmental}}\;\; {\mathbf{Impact}} & = 14.53 - 1.90A + 0.00B + 3.10 C + 0.25AB + 0.25BC \\ & \quad + 2.75AC + 1.68A^{2} + 1.18B^{2} + 0.68C^{2} \\ \end{aligned}$$

9 $$\begin{aligned} {\mathbf{Cost}} \;\;{\mathbf{effectiveness}} & = 22.39 + 2.96A + 0.79B + 0.61 C + 0.02AB + 0.08BC \\ & \quad + 0.12AC - 0.18A^{2} + 0.49B^{2} + 0.27C^{2} \\ \end{aligned}$$

### Impact of environmental parameters on performance outcomes of EPB

The environmental parameters, including Dry Bulb Temperature (DBT), Relative Humidity (RH), and Direct Normal Irradiance (DNI), play a pivotal role in shaping the performance outcomes of solar photovoltaic (PV) cells when coated with various biodegradable polymer materials. These parameters collectively represent the climatic conditions that PV cells may encounter in real-world applications, and their impact on performance outcomes is significant.

**Figure 2:**
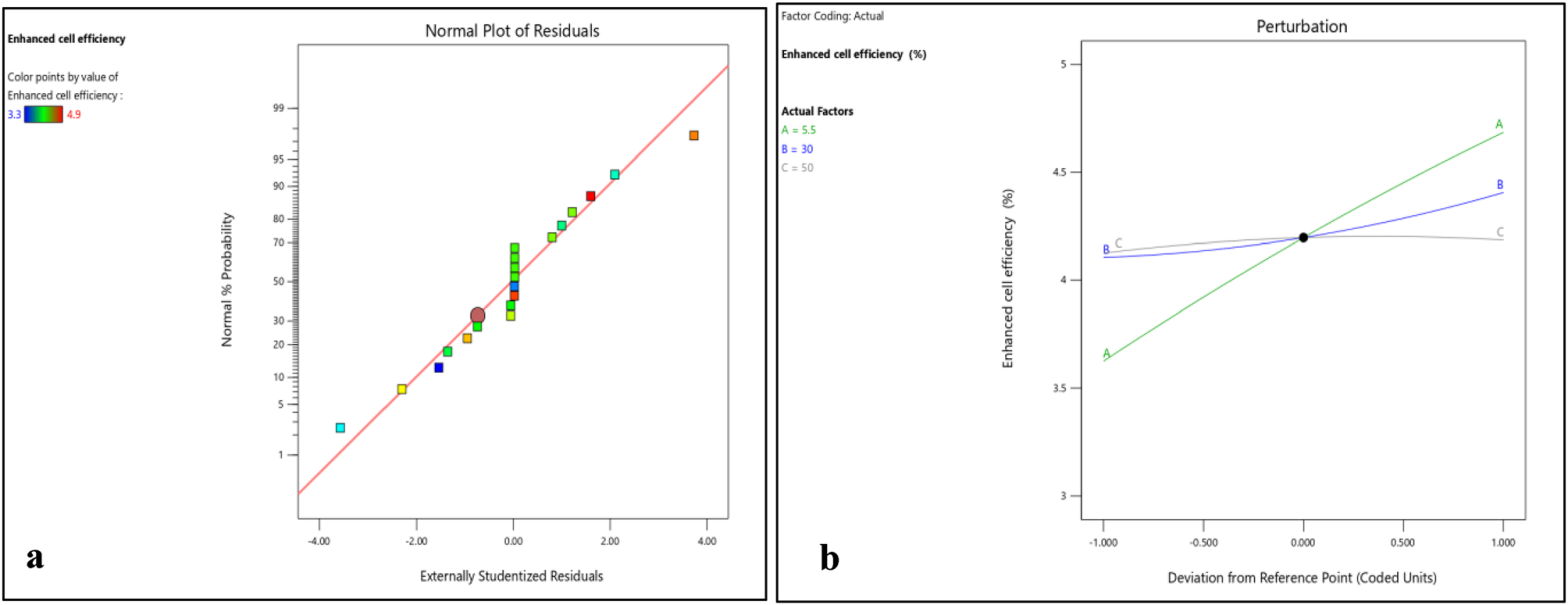
(**a**) Normality plot and (**b**) perturbation plot for enhanced cell efficiency.

**Figure 3:**
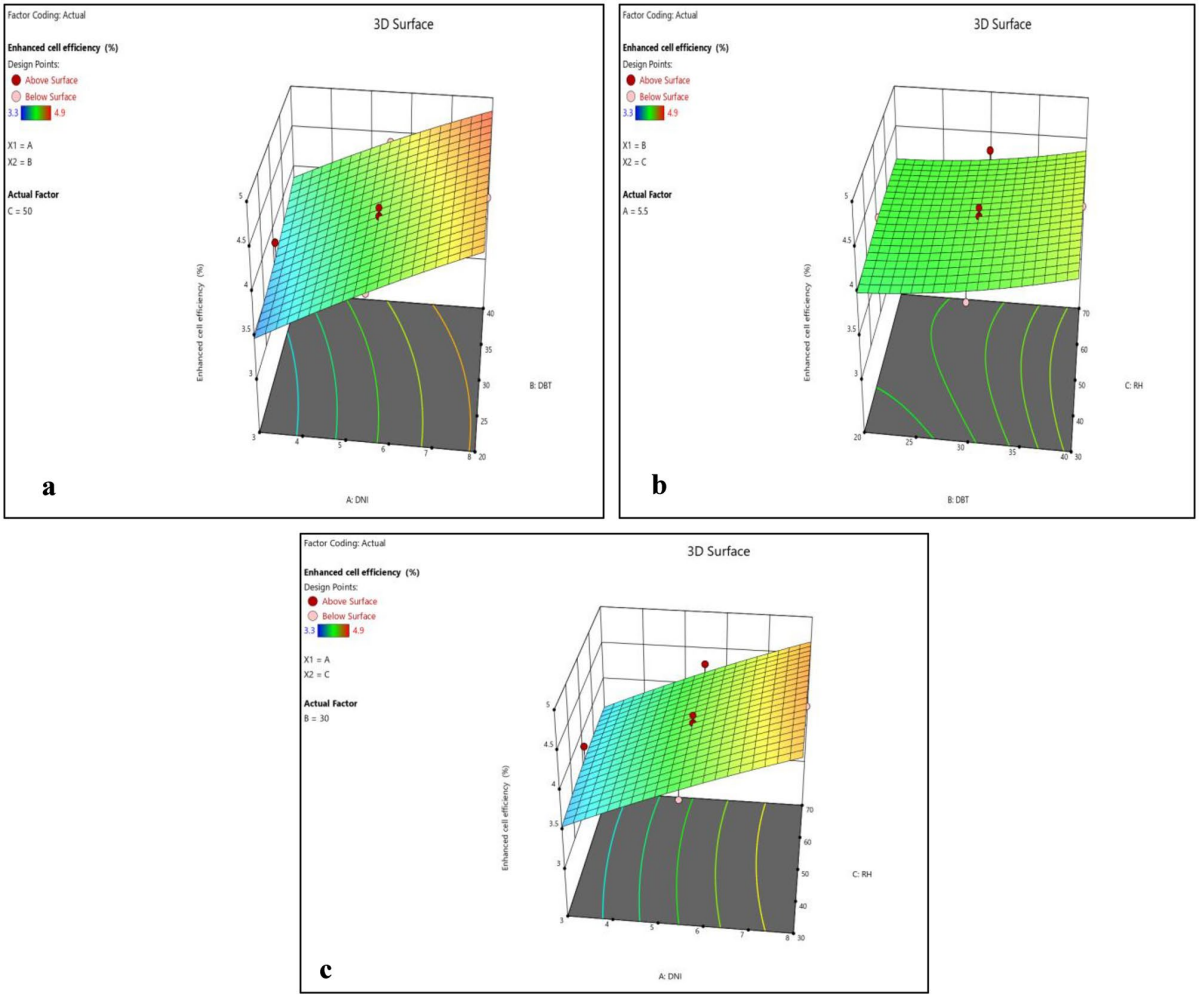
3D surface plots for enhanced cell efficiency.

**Figure 4:**
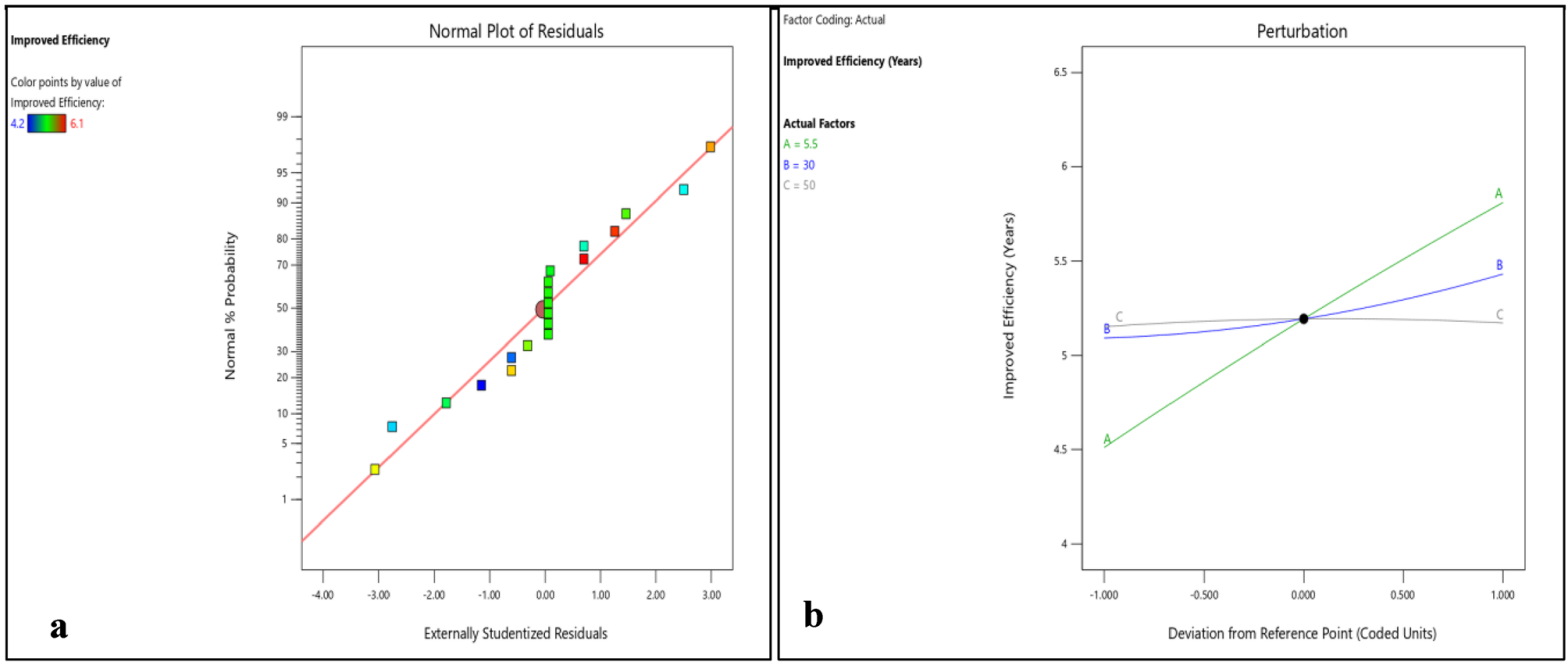
(**a**) Normality plot and (**b**) perturbation plot for improved durability.

**Figure 5:**
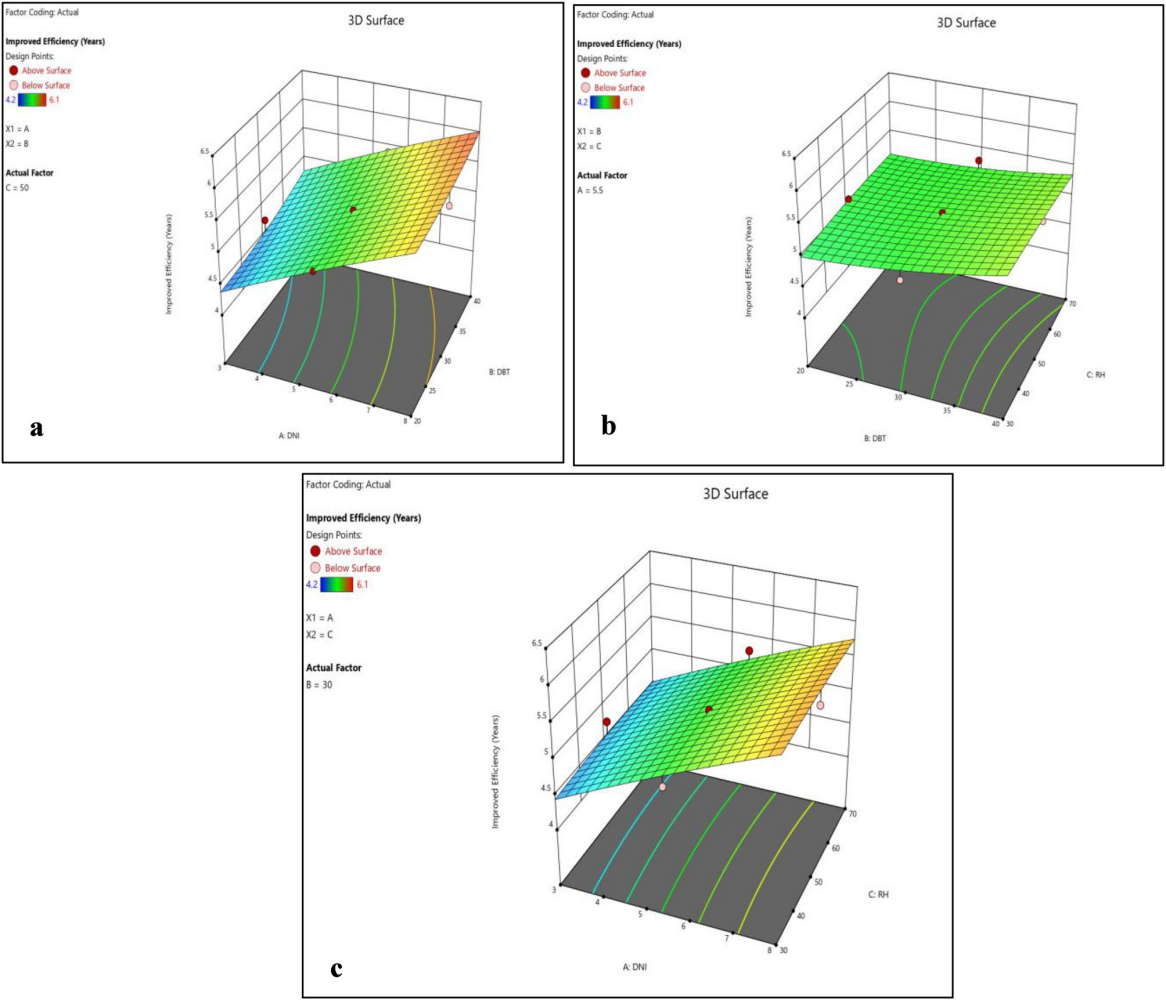
3D surface plots for improved durability.

**Figure 6:**
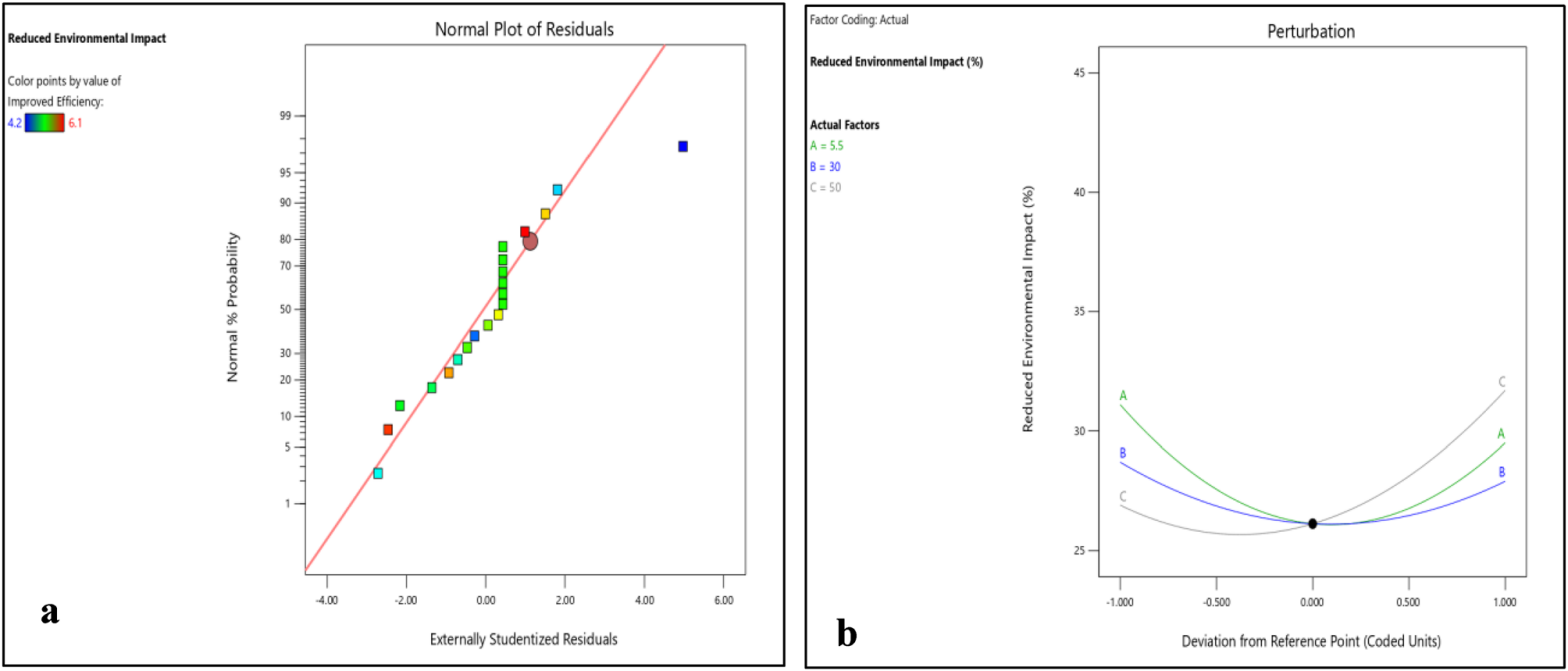
(**a**) Normality plot and (**b**) perturbation plot for reduced environmental impact.

**Figure 7:**
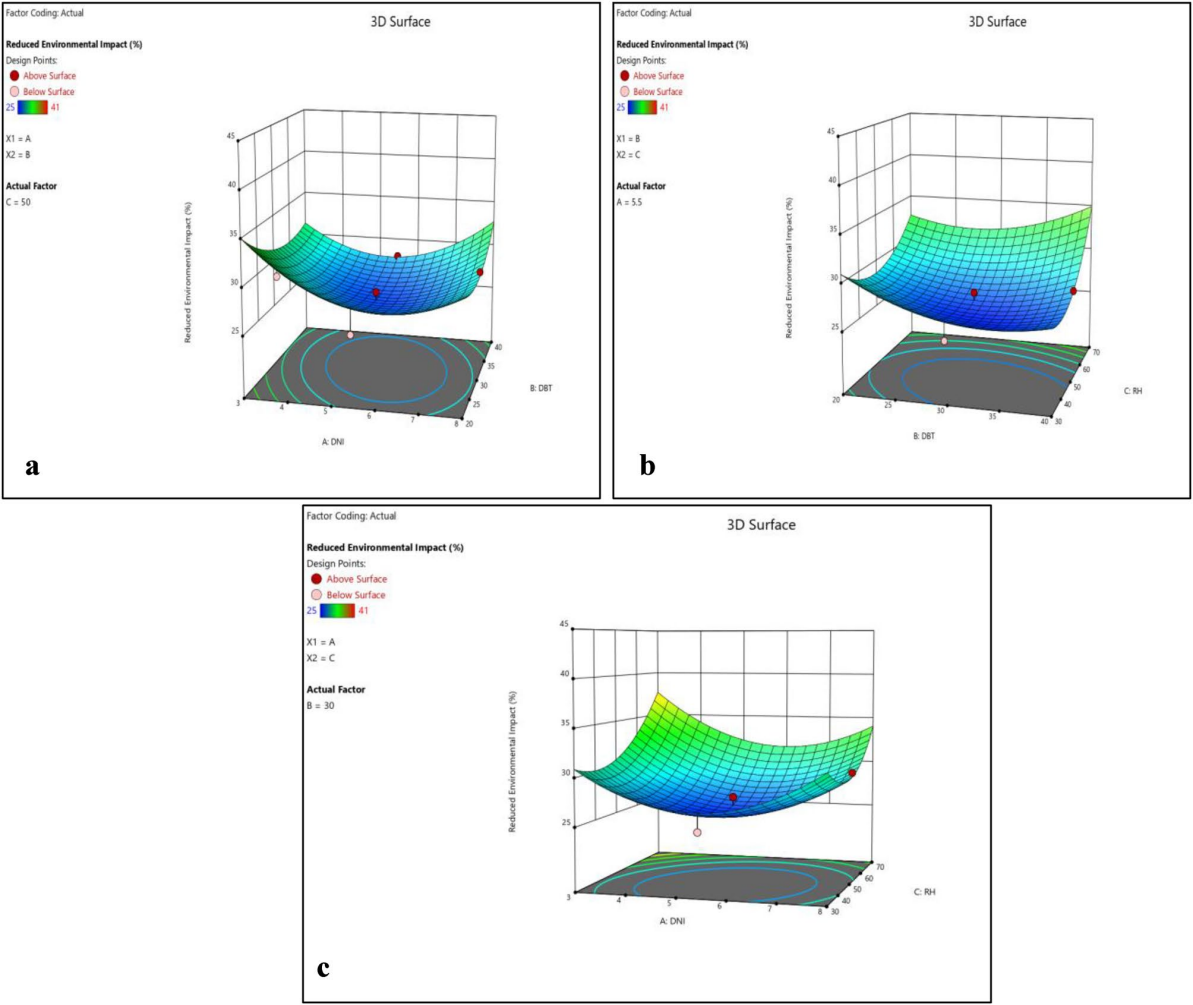
3D surface plots for reduced environmental impact.

**Figure 8:**
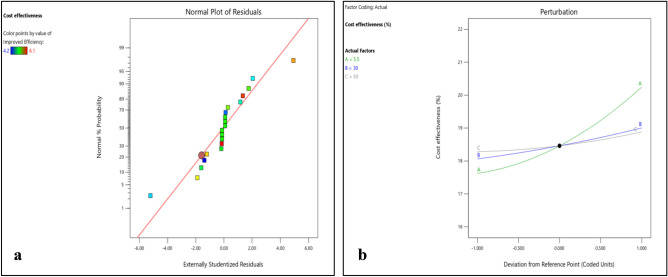
(**a**) Normality plot and (**b**) perturbation plot for cost effectiveness.

**Figure 9:**
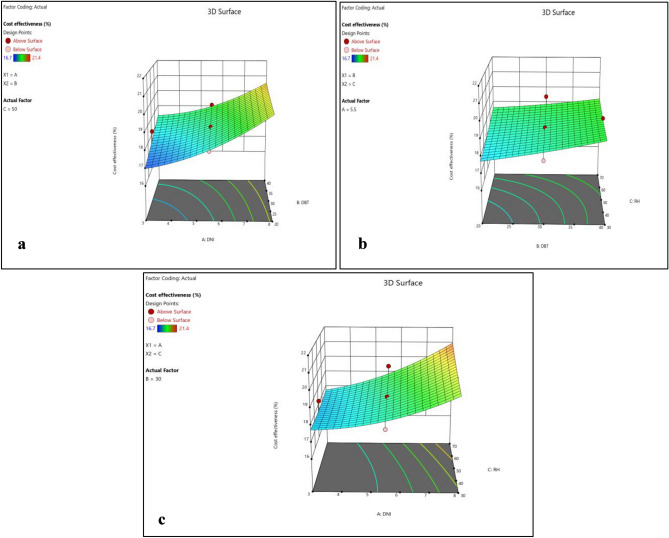
3D surface plots for reduced environmental impact.

Normality plots and 3D surface plots for all responses (i.e. enhanced cell efficiency, improved durability, reduced environmental impact and cost effectiveness) are presented in Figs. 2, 3, 4, 5, 6, 7, 8 and 9. For the sake of explanation all the interaction plots of different responses are presented. For every output response studied, it can be observed that the trend shows by perturbation plots corresponds to the same nature as explained in corresponding 3D surface plots. Normality plots of all responses shows that data points are nearer or on the centre line which indicates that the data are normally distributed. Perturbation plot and 3D surface plot for enhanced cell efficiency as presented in Figs. 2b, 3a–c shows that enhance cell efficiency increases with direct normal radiation (DNI). Small increase in enhanced cell efficiency was also observed with increase in dry bulb temperature (DBT). However, marginal variation in enhanced cell efficiency was observed with increase in relative humidity (RH). Higher DBT levels, such as 40 °C, tend to reduce the efficiency of PV cells. This decrease in efficiency can be attributed to increased thermal stress on the cells, affecting their ability to convert sunlight into electricity effectively.

Figures 4b and 5a–c shows that improved durability significantly increase with increase in DNI, Small increase in improved durability was also observed with increase in DBT however, insignificant variation in improved durability was observed with increase in RH. Moreover RH representing relative humidity, demonstrates a nuanced influence on performance outcomes. Higher RH levels, like 80%, tend to impact the durability of PV cells coated with biodegradable polymers, reducing their lifespan compared to lower RH levels.

Figures 6b and 7a–c shows that the variation in reduced environmental impact with DNI and DBT are of same nature. Reduced environmental impact first decreases with increase in DNI and DBT separately and then start increasing. However, with increase in RH it first decreases slightly and then steep rise in reduced environmental impact was observed. DNI has a significant impact on enhanced cell efficiency, directly affecting power output. PV cells coated with EPB demonstrate remarkable efficiency gains under higher DNI levels, reaching up to 10% increase.

Figure 8b and 9a–c shows that cost effectiveness significantly increase with increase in DNI, however, small increase in cost effectiveness was observed with increase in DBT and RH. However, variations in DNI have limited effects on durability, environmental impact reduction, and cost-effectiveness, suggesting that the choice of polymer material plays a more substantial role in these aspects. This highlights the suitability of EPB for regions with intense solar radiation, where maximizing efficiency is critical.

### Impact of environmental parameters on performance outcomes of NBCS

**Figure 10:**
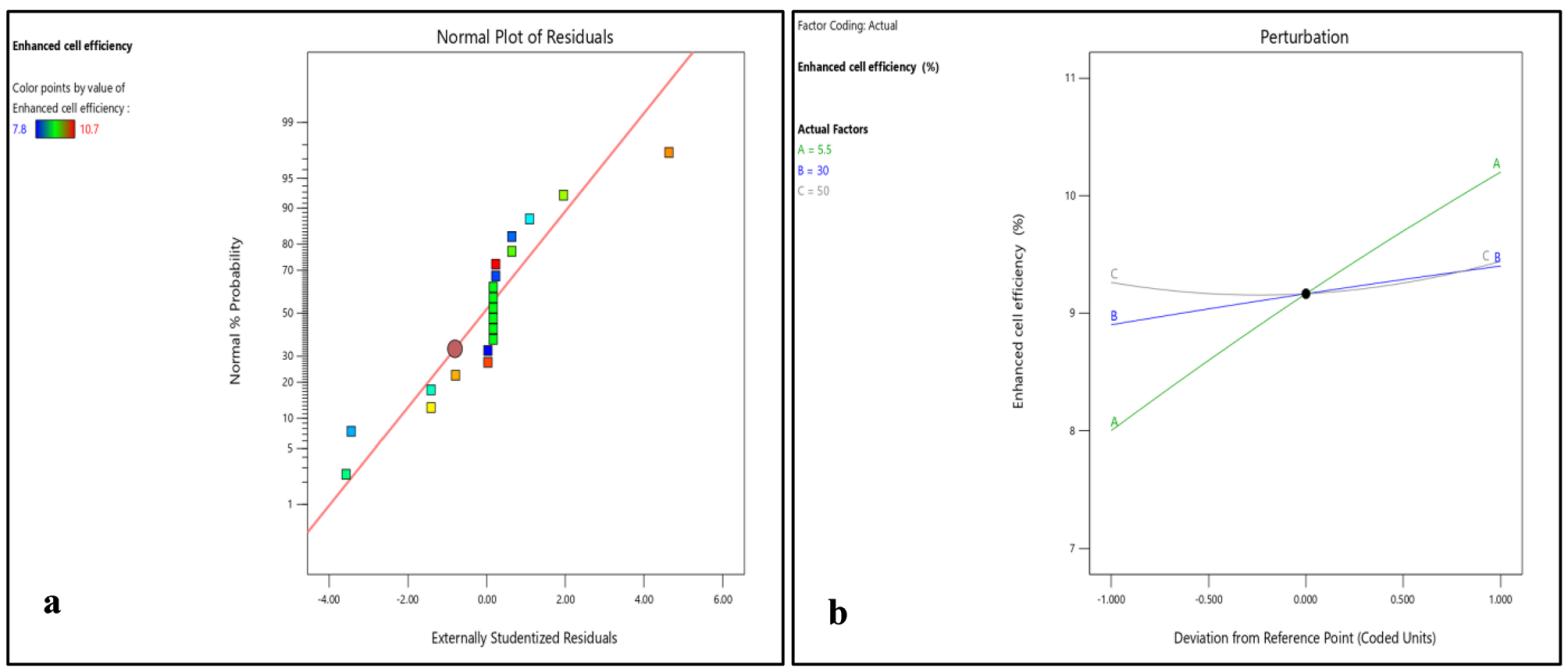
(**a**) Normality plot and (**b**) perturbation plot for enhanced cell efficiency.

**Figure 11:**
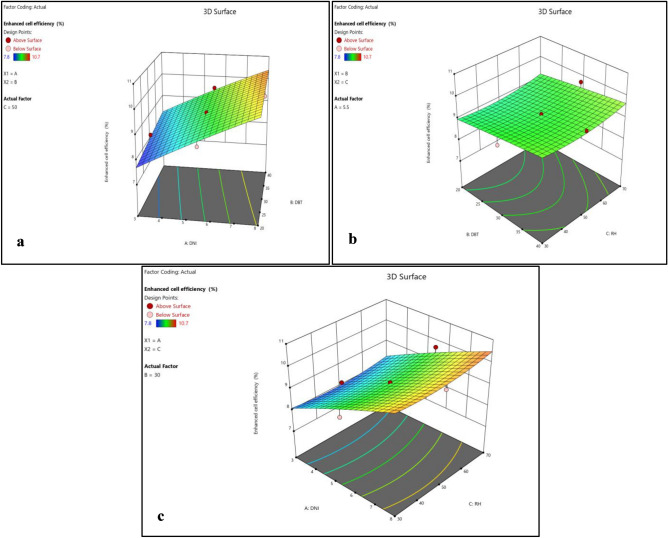
3D surface plots for enhanced cell efficiency.

**Figure 12:**
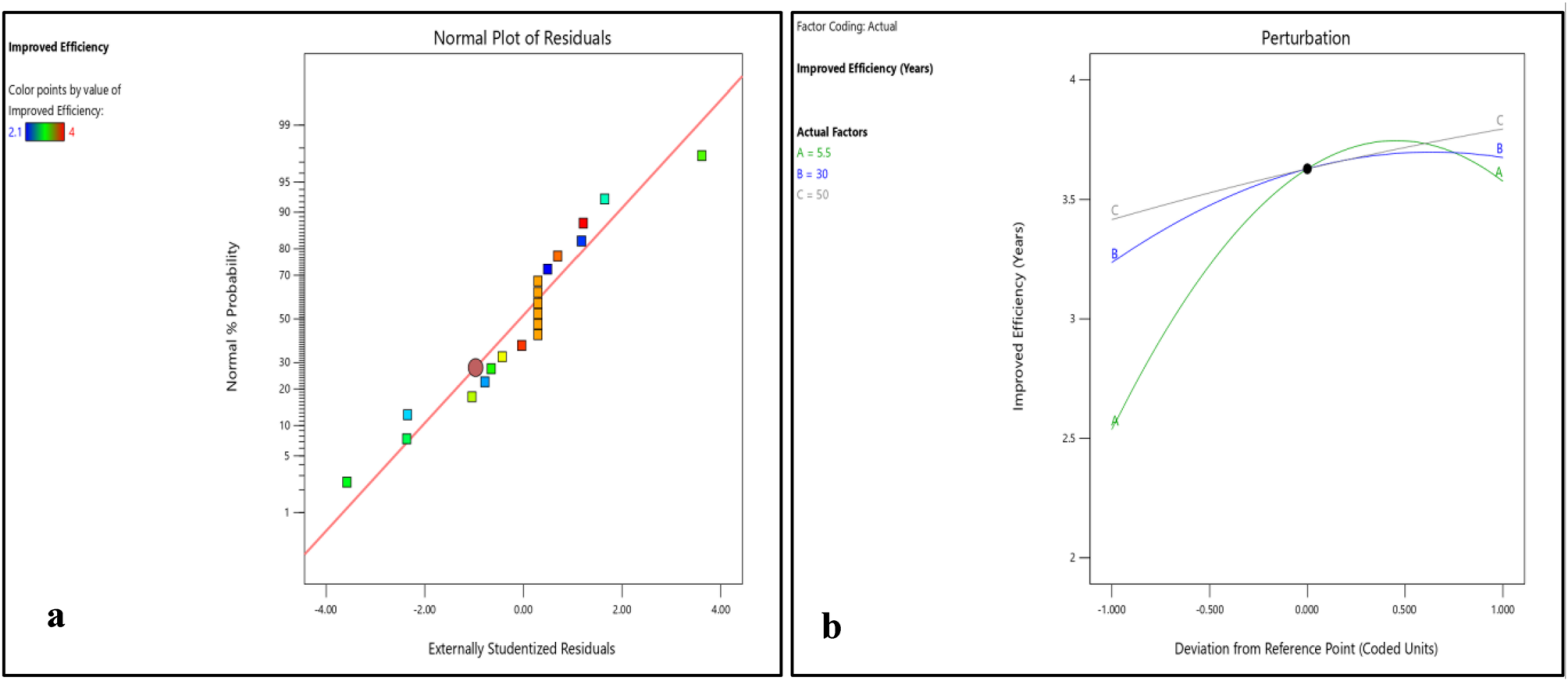
(**a**) Normality plot and (**b**) perturbation plot for improved efficiency.

**Figure 13:**
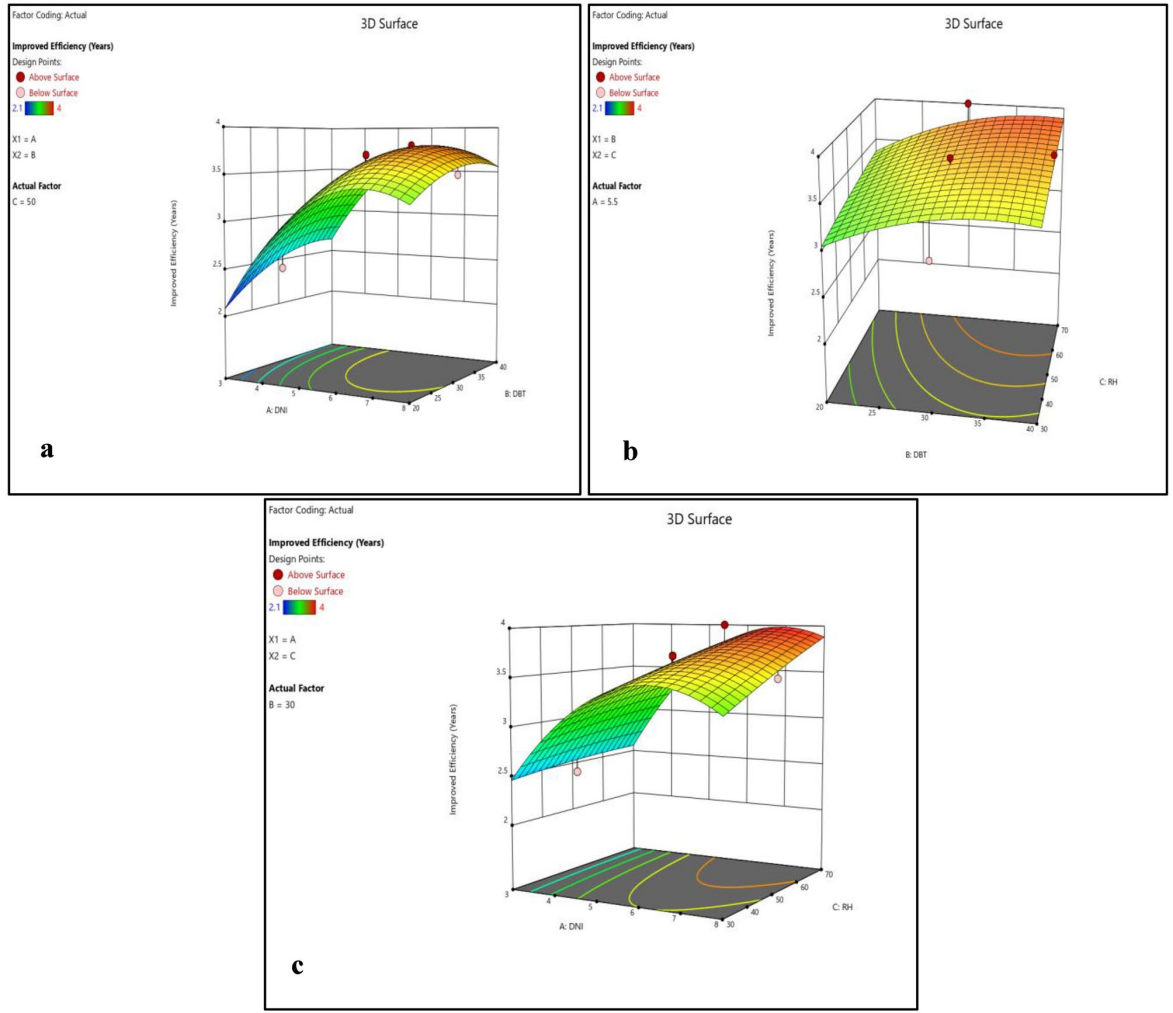
3D surface plot for improved efficiency.

**Figure 14:**
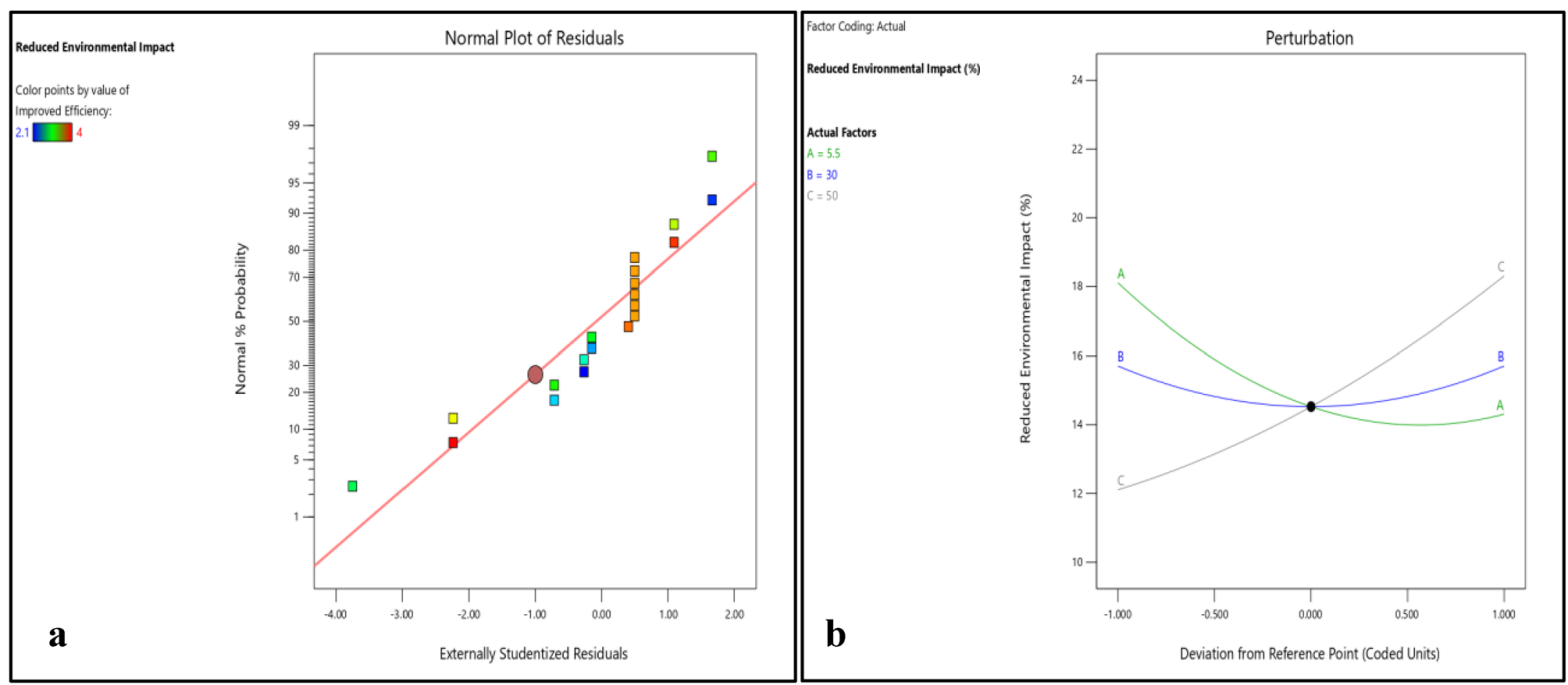
(**a**) Normality plot and (**b**) perturbation plot for reduced environmental impact.

**Figure 15:**
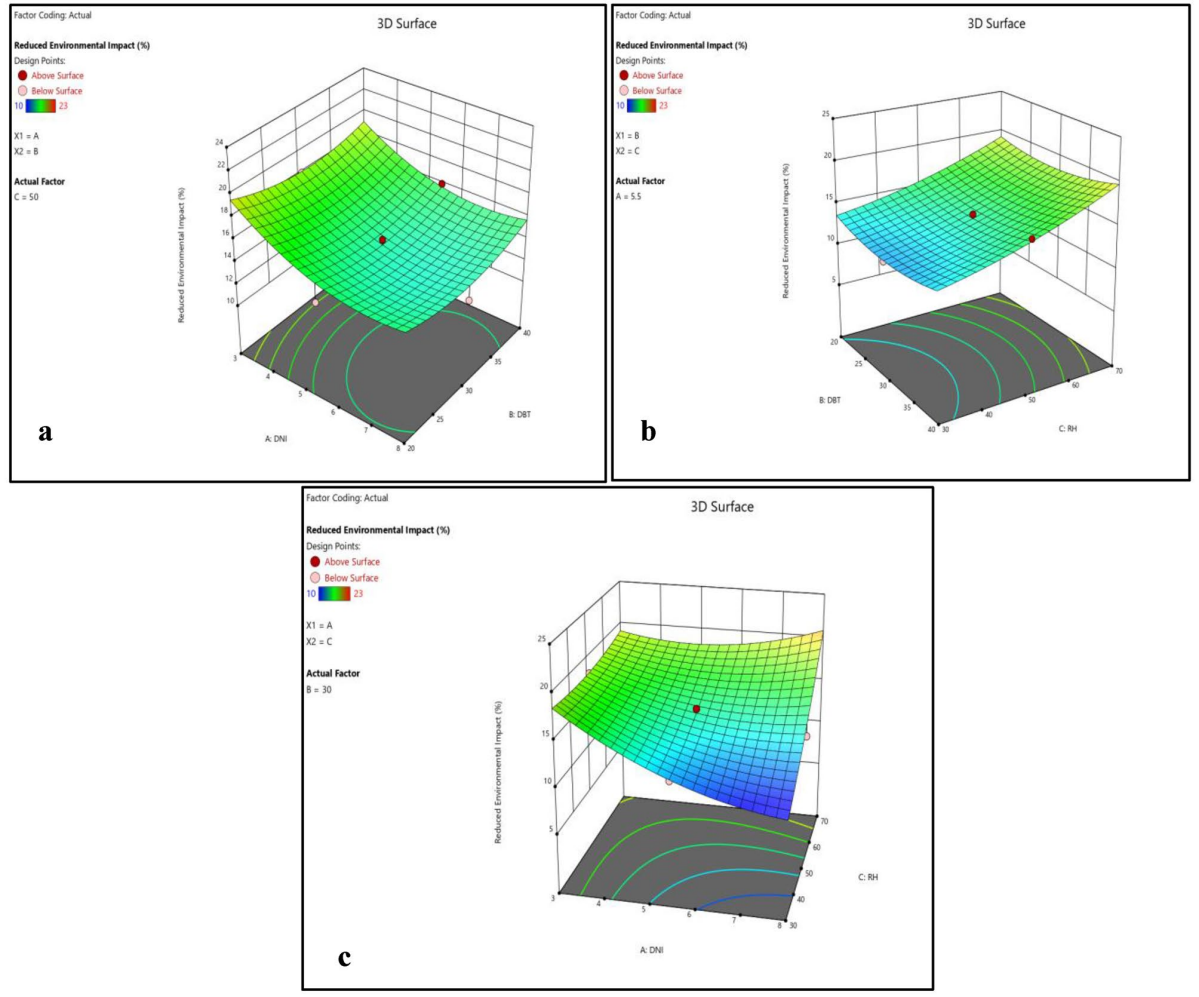
3D surface plot for reduced environmental impact.

**Figure 16:**
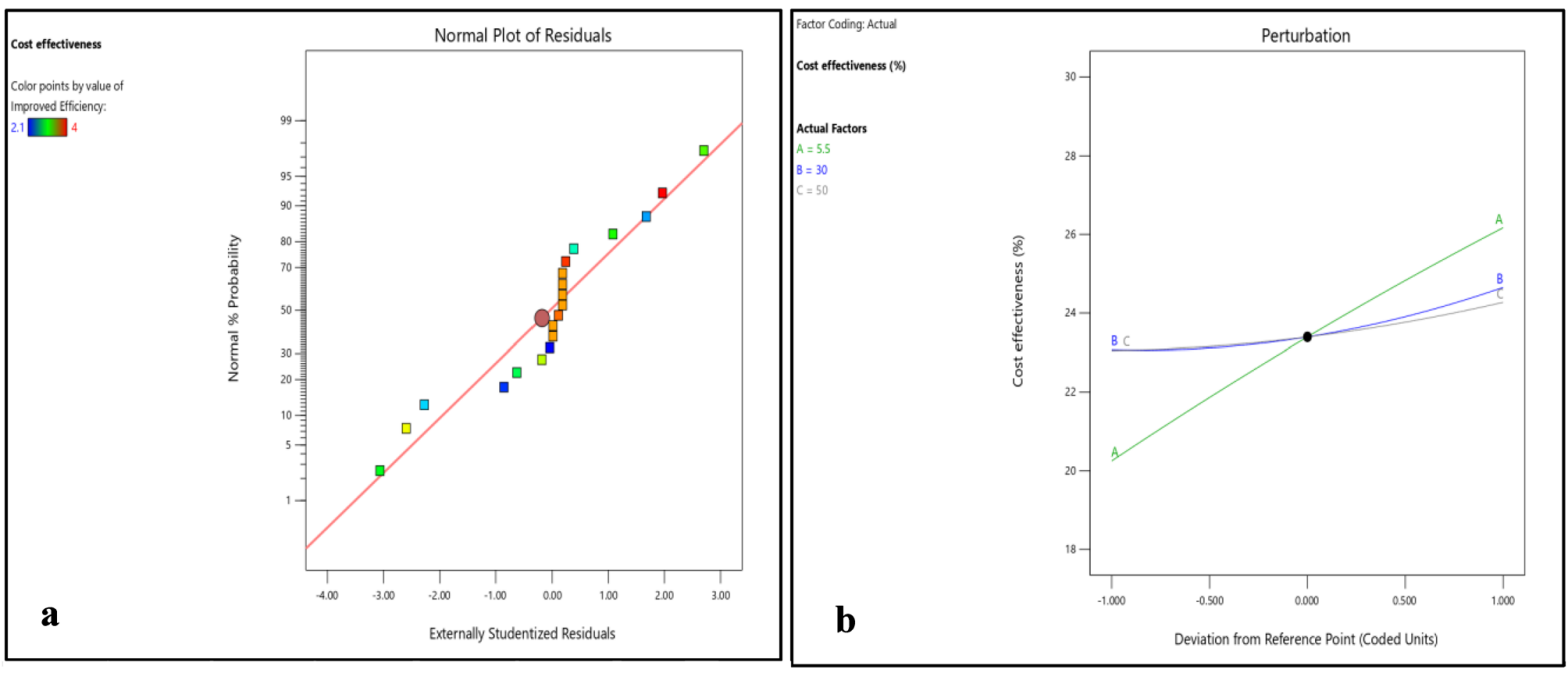
(**a**) Normality plot and (**b**) perturbation plot for cost effectiveness.

**Figure 17:**
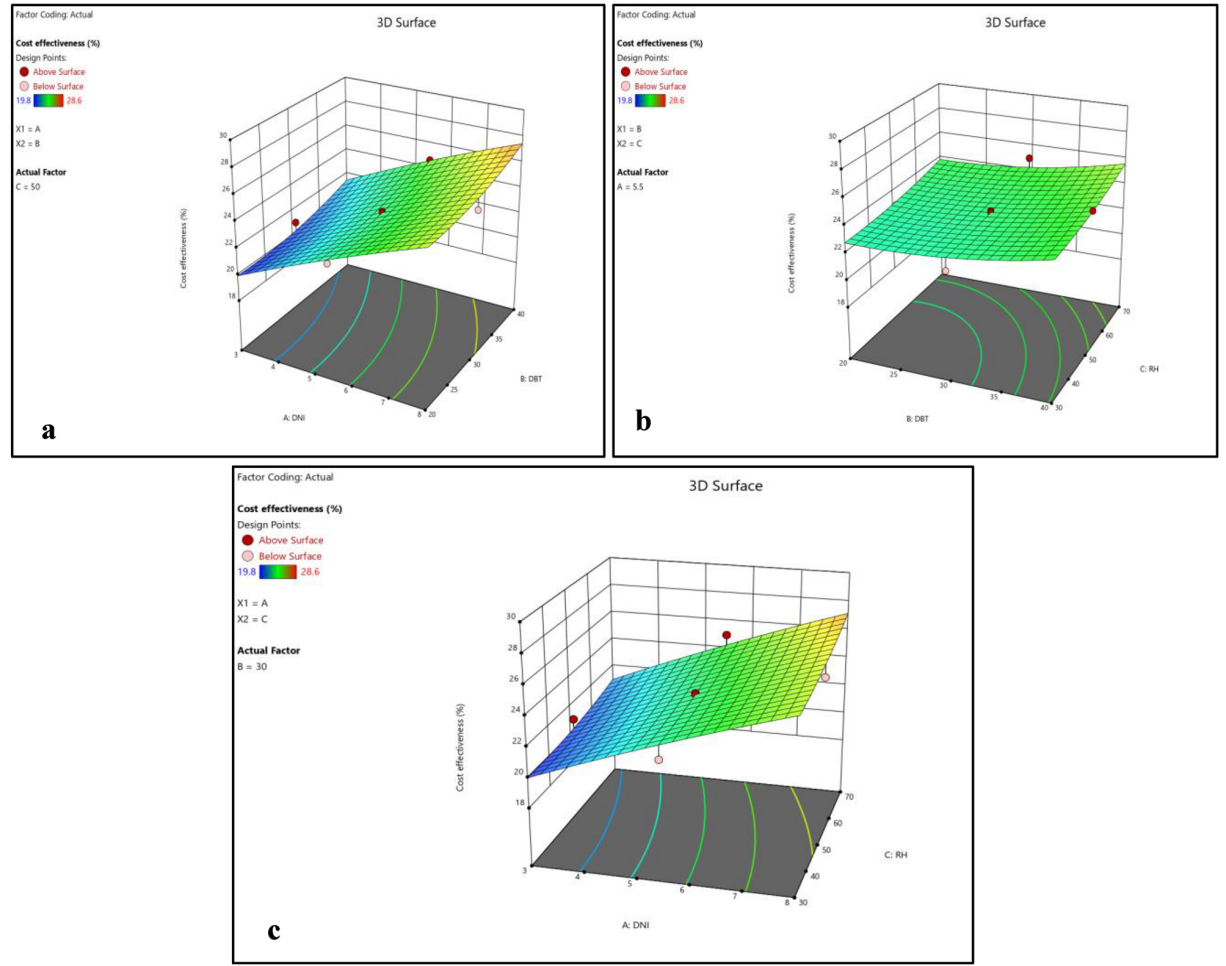
3D surface plot for cost effectiveness.

Normality plots and 3D surface plots for all responses (i.e. enhanced cell efficiency, improved durability, reduced environmental impact and cost effectiveness) are presented in Figs. 10, 11, 12, 13, 14, 15, 16 and 17. Here also all the interaction plots of different responses are presented. For every output response studied, it can be observed that the trend shows by perturbation plots corresponds to the same nature as explained in corresponding 3D surface plots. Normality plots of all responses shows that data points are nearer or on the centre line which indicates that the data are normally distributed. Perturbation plot and 3D surface plot for enhanced cell efficiency as presented in Figs. 10b and 11a–c shows that enhance cell efficiency significantly increases with direct normal radiation (DNI). Increase in enhanced cell efficiency was also observed with increase in dry bulb temperature (DBT). However, with increase in relative humidity (RH) enhanced cell efficiency marginally decreases in beginning and then small increment was observed. DBT, representing the ambient temperature, exerts a profound influence on PV cell efficiency. However, DBT variations have a less pronounced effect on the durability and cost-effectiveness of these materials.

Perturbation plot and 3D surface plot for improved durability was presented in Figs. 12b and 13a–c. It can be inferred from the figures that improved durability significantly increases with DNI in beginning but started to decrease towards last. Increase in improved durability was also observed with increase in DBT and RH. This is likely due to increased moisture exposure, which can degrade the materials over time. Additionally, higher Rh levels tend to favor reduced environmental impact by further decreasing the carbon footprint, aligning with sustainability goals.

Figures 14b and 15a–c shows that the reduced environmental impact decreases continuously with increase in DNI and was nearly constant towards end. With increase in DBT reduced environmental impact marginally decreases at beginning till mid-point then start increasing. However, with increase in RH steep rise in reduced environmental impact was observed. The effect of RH on enhanced cell efficiency varies among the polymer materials, emphasizing the need to consider material-specific interactions with humidity levels.

Figures 16b and 17a–c shows that cost effectiveness significantly increase with increase in DNI, however, small increase in cost effectiveness was observed with increase in DBT and RH. The interplay between environmental parameters and the type of biodegradable polymer material used for coating PV cells is complex and multifaceted. The study employs randomization techniques and carefully selected variable levels to minimize duplicate results and enhance reliability. Statistical methods such as ANOVA are utilized to identify genuine effects and mitigate duplication risks, ensuring the validity of findings through rigorous experimental design and analysis.

### Optimization

**Figure 18:**
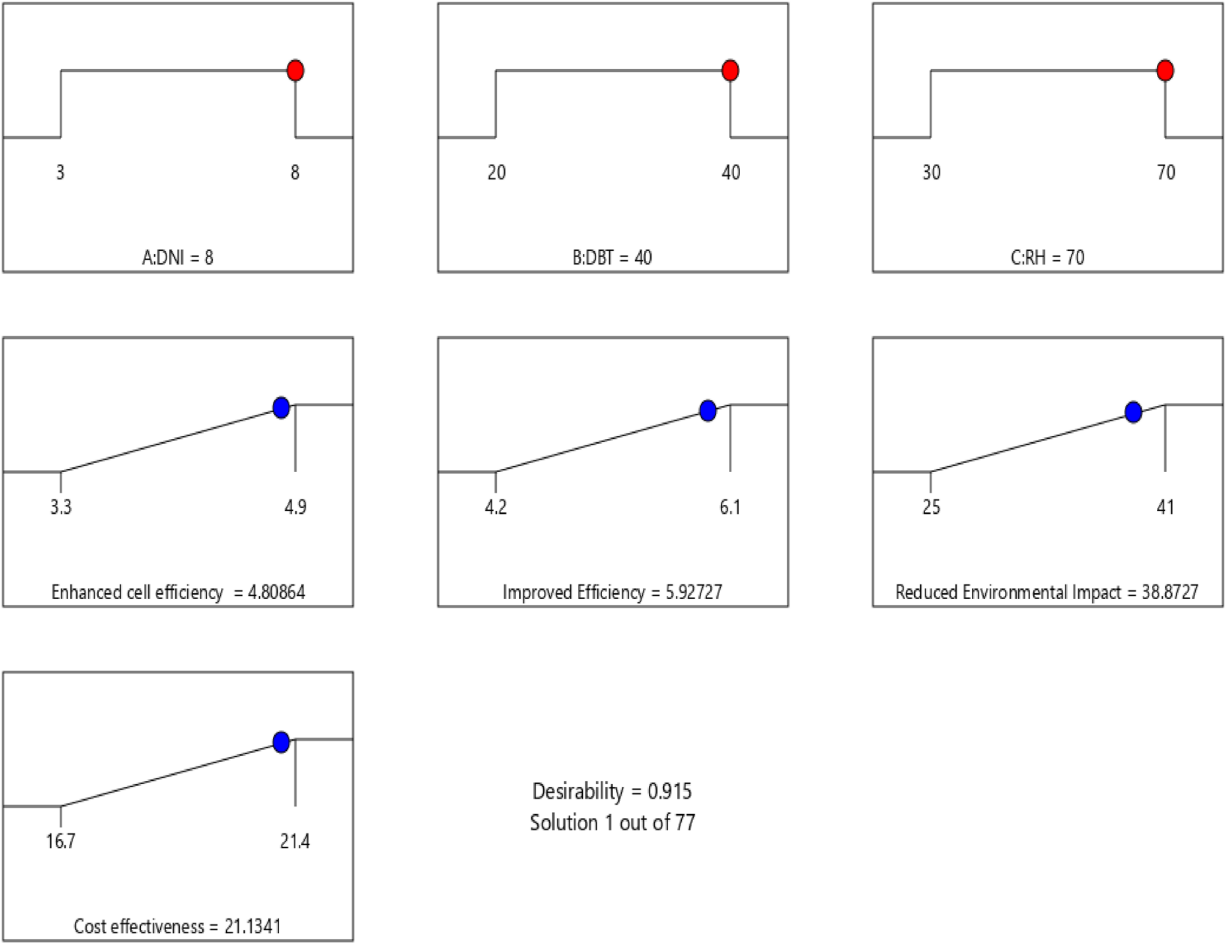
Desirability result for EPB coated material.

**Figure 19:**
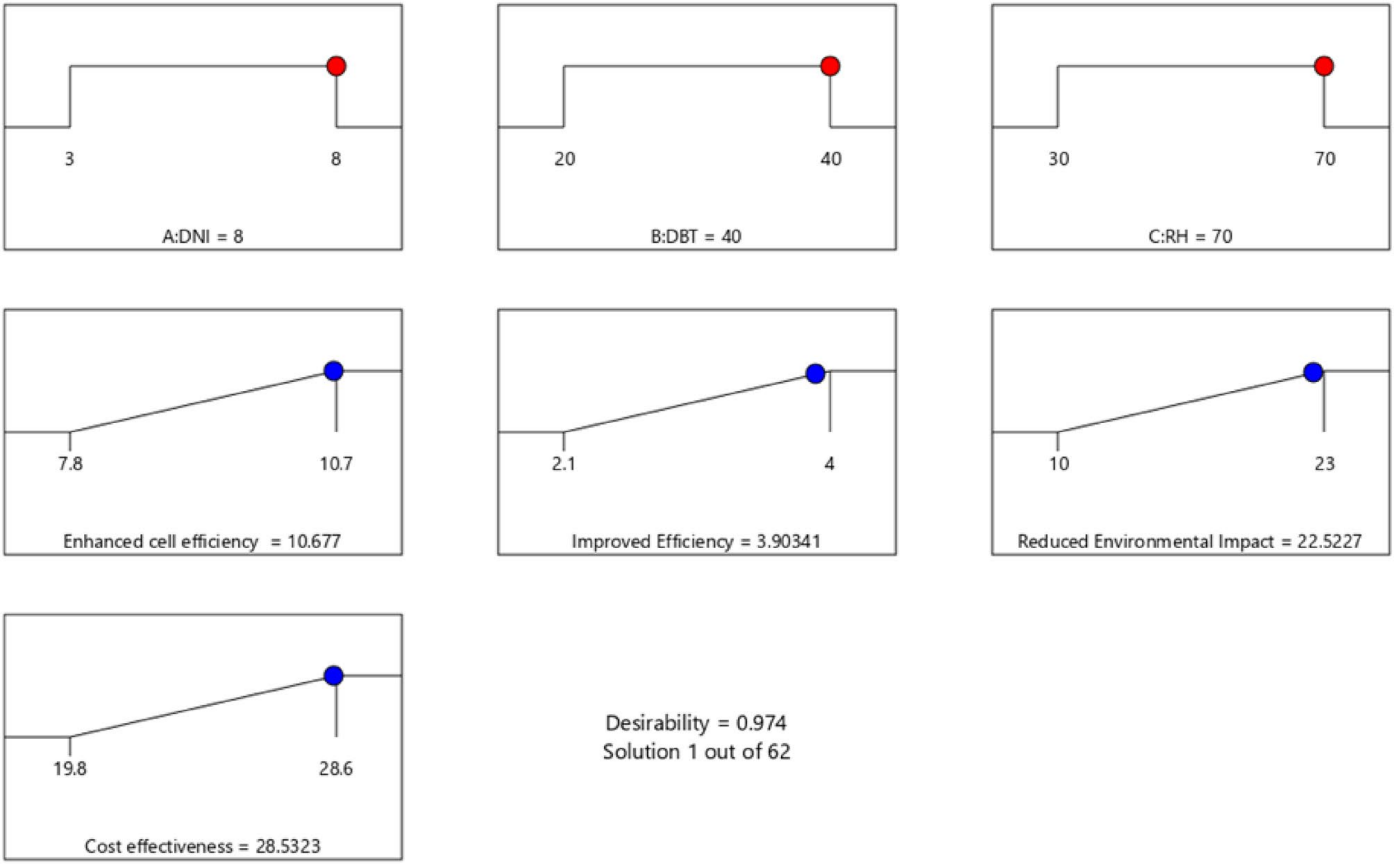
Desirability result for NBCS coated material.

Optimization of input parameters was done using desirability function approach. The result of the desirability is presented in Figs. 18 and 19 for EPB and NBCS coated material. The criterion of desirability function is to select the input parameter setting with maximum overall desirability. This methodology is used in other engineering problems also^41–44^. It is evident from Fig. 18 that the combined desirability value is 0.915 for EPB coated material which makes this model a well suited one for maximizing the responses. The optimized input parameter setting is DNI = 8 W/m^2^, dry bulb temperature, DBT = 40 °C and relative humidity = 70%. At the optimized parameter setting, enhanced cell efficiency = 4.8%, Improved durability = 5.9 Yrs, reduced environmental impact = 38.87% and cost effectiveness = 21.13%.

Figure 19 that the combined desirability value is 0.974 for NBCS coated material which makes this model a well suited one for maximizing the responses. The optimized input parameter setting is DNI = 8 W/m^2^, dry bulb temperature, DBT = 40 °C and relative humidity, RH = 70%. At the optimized parameter setting, enhanced cell efficiency = 10.67%, Improved durability = 3.90 Yrs, reduced environmental impact = 22.52% and cost effectiveness = 28.53%.

### Discussion

The results provides a comprehensive comparison of various types of materials used for enhancing the efficiency, durability, and environmental impact of solar cells. Biodegradable polymer blends offer a 2% increase in power output, making them a viable option for those seeking a modest boost in efficiency. This increase in power output is significant because even a small improvement can translate into substantial energy production gains over the long term, especially in large-scale solar installations. It means that for every 100 units of electricity produced by a standard solar cell, the use of these polymer blends results in an additional 2 units, which can make a considerable difference in energy generation. Their 5-year lifespan and 30% reduced carbon footprint contribute positively to sustainability. The 5-year lifespan is important because it strikes a balance between durability and the need for periodic maintenance or replacement. This reduces the overall environmental impact associated with solar cell production and disposal. The 30% reduction in carbon footprint signifies a significant decrease in greenhouse gas emissions during the materials' lifecycle, which aligns with global efforts to mitigate climate change and reduce our carbon footprint. Moreover, their cost-effectiveness, with a 20% lower cost per unit, makes them an attractive choice for those on a budget. This cost-effectiveness is crucial for both individual consumers and businesses looking to adopt solar technology. It allows for wider accessibility to solar energy, promoting its adoption on a broader scale and contributing to the transition towards cleaner and renewable energy sources. However, their relatively thick coating of 150 nm and susceptibility to UV radiation and temperature fluctuations may limit their applicability in certain environments. The thickness of the coating can affect the efficiency of light absorption and transmission, which is critical for solar cells. Additionally, the vulnerability to UV radiation and temperature fluctuations could lead to a reduction in the material's performance in regions with extreme weather conditions, such as deserts or high-altitude areas. Nevertheless, their ability to maintain 95% of initial efficiency after 10 years of exposure is commendable. This long-term performance is significant because it ensures that the material continues to deliver consistent power output over an extended period. It also minimizes the need for frequent replacements or maintenance, reducing the overall cost and environmental impact associated with solar cell systems. This durability can be particularly advantageous in regions where solar installations are subject to challenging environmental conditions or where maintenance is logistically difficult.

On the other hand, bio-based nanomaterials offer a substantial 10% increase in power output, making them a top choice for significantly improving cell efficiency. This remarkable increase in power output is of significant importance, as it can lead to significantly higher electricity generation from the same solar cell area. For industries and applications where maximizing energy production is critical, such as space-constrained installations or locations with high energy demand, this boost in efficiency can result in substantial cost savings and greater energy independence. However, their relatively short lifespan of 3 years and higher cost per unit offset some of these gains. The limited lifespan is a crucial factor to consider, as it implies that these materials may require more frequent replacement or maintenance. This can lead to increased downtime and overall operational costs, which might not be favorable in settings where long-term reliability and minimal disruption are essential. Additionally, the higher cost per unit can be a significant barrier to entry for some consumers or businesses, potentially limiting their adoption. Their 30% reduced carbon footprint is an environmental plus, aligning with sustainability goals and contributing to the reduction of greenhouse gas emissions. This reduction in carbon footprint can have a far-reaching impact, particularly in regions with a strong focus on environmental conservation and a commitment to mitigating climate change. It underscores the potential of bio-based nanomaterials to reduce the environmental impact of solar energy production. However, they require a thicker coating of 200 nm, which may impact their integration with solar cells. The increased coating thickness can hinder light transmission and absorption efficiency, potentially reducing the overall effectiveness of the solar cell. This factor is particularly relevant in situations where space is limited, and maximizing the number of solar cells in a given area is crucial. Nevertheless, these materials excel in high humidity conditions, extending the lifespan of solar cells by 8 years compared to uncoated cells. This enhanced durability in high humidity environments can be of immense significance, especially in regions prone to such conditions. It means that the investment in bio-based nanomaterials can lead to a more robust and long-lasting solar cell system, reducing the need for frequent replacements and maintenance. This benefit can be especially valuable in remote or challenging locations where access for maintenance may be limited. The study utilizes biodegradable polymers from anaerobic digestate to enhance PV cell performance and address environmental sustainability. By substituting conventional polymers with biodegradable alternatives, it reduces environmental footprint, promotes circular economy principles, and contributes to wider adoption of clean energy, aligning with climate change mitigation efforts.

### Validation of the results with previous literature

Desirability results for both the EPB coated and NBCS coated material showed that optimized parameter setting for both coated material is same i.e. DNI = 8 W/m^2^, dry bulb temperature, DBT = 40 °C and relative humidity, RH = 70%. However, on comparison of results it was concluded that for enhanced cell efficiency and cost effectiveness EPB coated material is more suited however for improving durability and reducing environmental impact NBCS coated material is more preferable choice as shown in Table 10.

**Table 10:** Validation of present study with earlier materials used.

S.No.	References	Materials	Enhanced cell efficiency	Improved durability	Environmental impact	Cost-effectiveness
1	Li et al.^9^	Biodegradable material	Increase		Eco-friendly	Low
2	Zhu et al.^15^	Wood	Increase	Increase	–	Low
3	Seck et al.^20^	Almond gum	–	–	Eco–friendly	Low
4	Amin et al.^23^	Paper/flexible substrates	No change	–	Eco–friendly	–
5	Dinh le et al.^19^	Graphene	Increase	–	Eco–friendly	Low
6	Current Study	EPB	Increase	Same	Eco–friendly	High
7	Current Study	NBCS	Increase	Increase	Eco–friendly	High

## Conclusions, limitations, policy making and future scope

### Conclusions

This study delves into the recovery and application of biodegradable polymers sourced from biomass anaerobic digestate with the aim of enhancing the performance of solar photovoltaic (PV) cells while championing environmental sustainability. Typically regarded as waste, the organic residues produced through the anaerobic digestion process are rich in biodegradable materials. However, this research endeavors to explore the potential of repurposing these materials by recovering and converting them into high-quality coatings or encapsulants for PV cells. These repurposed biodegradable polymers not only bolster the efficiency and longevity of PV cells but also contribute to sustainability objectives by curbing the carbon footprint associated with PV cell production and lessening environmental impact. Employing a comprehensive experimental design, the study varies coating thickness, direct normal irradiance (DNI) (A), dry bulb temperature (DBT) (B), and relative humidity (C) levels to analyze the interactions of various types of recovered biodegradable polymers with diverse environmental conditions. The following conclusions have been drawn from the study:

- Significance of parameters and interactions was established using ANOVA analysis.
- The developed model and response surface equations are observed to be highly suitable for predicting the required result.
- Optimization showed that better result was achieved at DNI = 8 W/m^2^, DBT = 40 °C and RH = 70% for both the coated material studied.
- Comparative study showed that for enhanced cell efficiency and cost effectiveness, EPB coated material is more suited.
- However for improving durability and reducing environmental impact NBCS coated material is a preferable choice.

### Policy making

Implementing policies to support the use of biomass anaerobic waste as coatings for solar panels requires a holistic approach that balances environmental benefits with potential challenges. One policy recommendation is to incentivize the adoption of waste-derived coatings through subsidies or tax incentives for renewable energy projects incorporating these materials. Governments can also promote research and development in this field by allocating funding and resources to drive innovation in sustainable coating technologies. However, a potential challenge lies in ensuring the safety and environmental compliance of these coatings, necessitating rigorous testing and certification procedures to verify their suitability for widespread use. Additionally, policies should address waste management regulations and standards to prevent any unintended environmental consequences that might arise from increased digestion and coating activities, particularly the proper disposal and treatment of residual waste.

Another critical aspect of policy-making involves setting standards and guidelines for the quality and performance of digestate-based coatings, including their durability and effectiveness in varying environmental conditions. Ensuring transparency in the manufacturing and application processes of these coatings is vital to building trust among consumers and stakeholders. Nevertheless, one potential hurdle is striking a balance between environmental protection and cost-effectiveness, as stringent regulations and standards might raise production costs and limit the scalability of this technology. Policymakers must carefully assess the trade-offs and design policies that foster innovation while safeguarding the environment. Ultimately, a well-crafted policy framework can encourage the adoption of biomass anaerobic waste coatings, driving sustainable practices in the solar energy sector while addressing the challenges that may arise along the way.

### Limitations of the study

The study explores using biomass anaerobic waste as solar panel coatings, yet acknowledges the need for further validation of their efficacy and long-term performance. Variability in feedstock composition and coating production methods, along with potential environmental impacts and scalability challenges, underline the necessity for comprehensive assessments and careful consideration of limitations in assessing their viability.Please note that the Tables are renumbered to ensure sequential ordering. Also the citations are changed in text.correct

### Future scope

The present study lays the groundwork for an exciting array of future research opportunities. One promising avenue is the optimization of biomass anaerobic waste coatings for solar panels, delving deeper into fine-tuning the composition and application methods to enhance their efficiency and longevity. Exploring alternative sources of anaerobic waste, such as agricultural residues or wastewater byproducts, offers potential variations and broader applications for these coatings. Moreover, comprehensive environmental impact assessments, including life cycle analyses, could provide a more holistic understanding of the sustainability benefits of these coatings. Collaborative efforts between researchers, policymakers, and industry stakeholders are vital to developing standardized guidelines and regulatory frameworks that facilitate the widespread adoption of waste-derived coatings, paving the way for a greener and more sustainable future in the renewable energy sector.Table 9 was not cited in text. Please confirm the citation as provided now is okay.okay

## Data Availability

The datasets used and/or analysed during the current study available from the corresponding author on reasonable request.
